# Development of Silica-Based Biodegradable Submicrometric Carriers and Investigating Their Characteristics as in Vitro Delivery Vehicles

**DOI:** 10.3390/ijms21207563

**Published:** 2020-10-13

**Authors:** Mikhail V. Zyuzin, Dingcheng Zhu, Wolfgang J. Parak, Neus Feliu, Alberto Escudero

**Affiliations:** 1 Department of Physics and Engineering, ITMO University, Lomonosova 9, St. Petersburg 191002, Russia; mikhail.zyuzin@metalab.ifmo.ru; 2 Center for Hybrid Nanostructures (CHyN), Universität Hamburg, 22607 Hamburg, Germany; dzhu@physnet.uni-hamburg.de (D.Z.); wolfgang.parak@uni-hamburg.de (W.J.P.); 3 Fraunhofer Center for Applied Nanotechnology (CAN), 20146 Hamburg, Germany; 4 Departamento de Química Inorgánica, Facultad de Química, Universidad de Sevilla, Calle Profesor García González 1, E–41012 Seville, Spain; 5 Instituto de Investigaciones Químicas (IIQ), Universidad de Sevilla–CSIC, Calle Américo Vespucio 49, E–41092 Seville, Spain

**Keywords:** silica capsules, gene delivery, endo/lysosomal escape, transfection, DNA

## Abstract

Nanostructured silica (SiO_2_)-based materials are attractive carriers for the delivery of bioactive compounds into cells. In this study, we developed hollow submicrometric particles composed of SiO_2_ capsules that were separately loaded with various bioactive molecules such as dextran, proteins, and nucleic acids. The structural characterization of the reported carriers was conducted using transmission and scanning electron microscopies (TEM/SEM), confocal laser scanning microscopy (CLSM), and dynamic light scattering (DLS). Moreover, the interaction of the developed carriers with cell lines was studied using standard viability, proliferation, and uptake assays. The submicrometric SiO_2_-based capsules loaded with DNA plasmid encoding green fluorescence proteins (GFP) were used to transfect cell lines. The obtained results were compared with studies made with similar capsules composed of polymers and show that SiO_2_-based capsules provide better transfection rates on the costs of higher toxicity.

## 1. Introduction

Nanostructured materials are widely applied in the field of drug and gene delivery [1,2,3]. For an effective drug administration, an appropriate design of the material providing the delivery of various bioactive compounds to the site of interest, a sufficient release profile, an improved solubility of drugs, among others, is demanded [4]. Drug carriers can be either based on organic (polymers, lipids) [5,6] or inorganic (gold, silver, iron oxide nanoparticles) [7,8] materials, or based on a combination of both [9,10]. Physicochemical properties of the developed drug vehicles, such as size, shape, and composition, regulate their interaction with cells and, consequently, their further fate in vitro and in vivo [11,12,13]. The whole field, however, suffers from the fact that carriers are in general endocytosed, and thus the delivered bioactive compounds remain trapped in endosomes/lysosomes [14]. Even when the exact mechanism for the cargo release is still unclear, there are attempts to enable release to the cytosol, for example, by chemical [15] of physical stimuli [16]. Such triggered release, in general, is not efficient, or induces toxicity [17,18,19]. In previous work, we have shown that upon hydrolysis SiO_2_-based capsules may rupture endo/lysosomal membranes after endocytosis, and thus warrant for the release of the encapsulated bioactive compounds to the cytosol of cells [20]. These capsules, however, were micrometer-sized, and there was no detailed assessment of the delivery efficiency and the toxicity, which is the subject of the present report.

Silica (SiO_2_)-based carriers of bioactive compounds attract attention due to their biocompatibility and a relatively low toxicity [21,22]. It is worth mentioning that here we consider amorphous SiO_2_ carriers formed in Stöber sol–gel reactions [23,24]. The porosity of SiO_2_-based structures can protect sensitive cargo from premature enzymatic degradation [25]. Indeed, due to the increased surface area and surface reactivity of SiO_2_ particles, different bioactive molecules can be adsorbed onto them by either the formation of H-bonds or due to London dispersion interactions [26]. Besides that, nanostructured SiO_2_ drug carriers are biodegradable at longer incubation times, and can be cleared from the body after several days [27]. The product of silica degradation is silicic acid, which is reported to act as a source of silicon (Si) for the formation of connective tissues [28]. Safety concerns of SiO_2_-based carriers are an issue [29,30], but the material compares well to other, more toxic carriers [31,32]. Another advantage of SiO_2_–based delivery platforms is the availability of low-cost synthesis methods that commonly take place in aqueous media and follow a sol-gel approach [33]. Such conventional approach for the formation of SiO_2_ particles includes the hydrolysis of various silanes or silicates with a subsequent SiO_2_ condensation [34]. This synthesis method allows for an accurate control of the physicochemical properties of the SiO_2_ particles such as size, shape, porosity, and others [35,36].

In order to increase the loading capacity of SiO_2_ -based carriers, SiO_2_ can be condensed and/or deposited onto sacrificial templates forming micellar structures [37]. For example, cetyltrimethylammonium bromide (CTAB) is a common surfactant molecule that is usually used for the further SiO_2_ deposition [38], though due to its toxicity this is not a favorable approach for biological applications [39,40]. Inorganic nano- and microparticles can be also used as templates for the formation of hollow SiO_2_-particles [41,42]. Among the latter, calcium carbonate (CaCO_3_) is often used as a core material for the SiO_2_ deposition [37], since CaCO_3_ is biocompatible and can be dissolved in mild conditions. The size of the CaCO_3_ particles determines the size of the resulting hollow SiO_2_ carries [36]. Although a large number of studies on the synthesis of SiO_2_-based drug delivery platforms can be found in the literature, there is still an increased interest in the developing of biocompatible SiO_2_ carriers with increased loading capacity, and also versatile in terms of encapsulated cargo, with the possibility of multiple drug loading. Moreover, comprehensive degradation studies of such carriers in biological fluids, as well as quantitative biological characterization in terms their performance, are also required.

In this work, we describe the synthesis of submicrometric SiO_2_ hollow capsules as carriers for biological molecules. The synthesis is based on the deposition of a SiO_2_ shell onto sacrificial CaCO_3_ templates that were further dissolved under mild conditions. Tetraethyl orthosilicate (TEOS) was used as a SiO_2_ source, and was added at different amounts to tune the SiO_2_ shell thickness. Different bioactive compounds were loaded into the sumicrometric cavity of the capsules, including dextran, proteins (DQ-OVA), and genetic material (deoxyribonucleic acid/DNA plasmids encoding green fluorescence protein/GFP) demonstrating the versatility of the developed carriers. For a comparative study, submicrometric capsules comprised of either synthetic or natural polymers were also synthesized [43].

**Figure 1:**
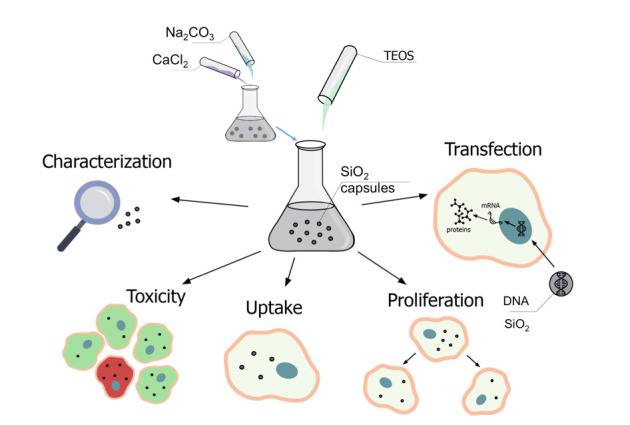
Road map of implemented steps of synthesis, characterization, and biological application of SiO_2_-based delivery.

The characterization of the submicrometric capsules of the different materials was performed using scanning electron microscopy (SEM), transmission electron microscopy (TEM), laser scanning confocal microscopy (CLSM), and dynamic light scattering (DLS). Moreover, the influence of biological fluids on the carriers and the encapsulated cargo was tested. Uptake by cell lines, and the effect on cell viability and proliferation of the obtained carriers was investigated with human cervical carcinoma (HeLa) cells. The SiO_2_-based submicrometric carriers were used to deliver DNA plasmids encoding GFP into cells at different incubation conditions, and the transfection efficiency was determined and compared with their polymer-based counterparts. Finally, the mechanisms of the improved transfection efficiency of SiO_2_-based submicrometric carriers were discussed. A schematic illustration of the performed steps in this study is depicted in Figure 1.

## 2. Results and Discussion

The main applied methodologies as used in this study are highlighted in Figure 1, and include the synthesis and characterization of submicrometric capsules (either SiO_2_ or polyelectrolyte-based), their biological characterization (including cellular uptake, effect on cell viability and proliferation, and degradation), and the delivery of DNA plasmids into HeLa cells, which was estimated using CLSM and flow cytometry.

### 2.1. Synthesis, Characterization, and Loading of Submicrometric Carriers Based on SiO_2_ and Polyelectrolytes

Submicrometric capsules loaded with different molecular cargos were synthesized by modifying the well-established approach based on the precipitation of CaCO_3_ cores in presence of the molecular cargo material (the so-called preloading strategy) [36,44]. The cores were further coated with either SiO_2_ or polyelectrolytes using the layer-by-layer (LbL) technique, and were finally dissolved yielding hollow capsules [36,43]. The used strategy to achieve smaller capsule sizes in comparison to previous protocols consisted of the precipitation of the salts (CaCl_2_ and Na_2_CO_3_) in a mixture of ethylene glycol (EG) and water [20]. To obtain a SiO_2_ shell around the particles, the CaCO_3_ cores were firstly stabilized with ɑ-methoxy-ω-mercapto poly(ethylene glycol) (CH_3_O-PEG-SH), and a further SiO_2_ layer was deposited onto the cores in ethanol by adding tetraethyl orthosilicate (TEOS) and ammonia. Different TEOS amounts (nominally low and high TEOS) were used with the aim of controlling the capsules' wall thickness. A final layer of poly(arginine) (PARG) was deposited onto the SiO_2_-based capsules to render the capsule surface positive charge. Polyelectrolyte-based capsules with two different architectures (biodegradable dextran sulfate/poly(arginine) (DEXS/PARG)_4_ and non-biodegradable polystyrene sulfonate/poly(allylamine hydrochloride) (PSS/PAH)_4_) were also synthesized using the LbL deposition approach on the submicrometric CaCO_3_ template cores [45]. 

**Figure 2:**
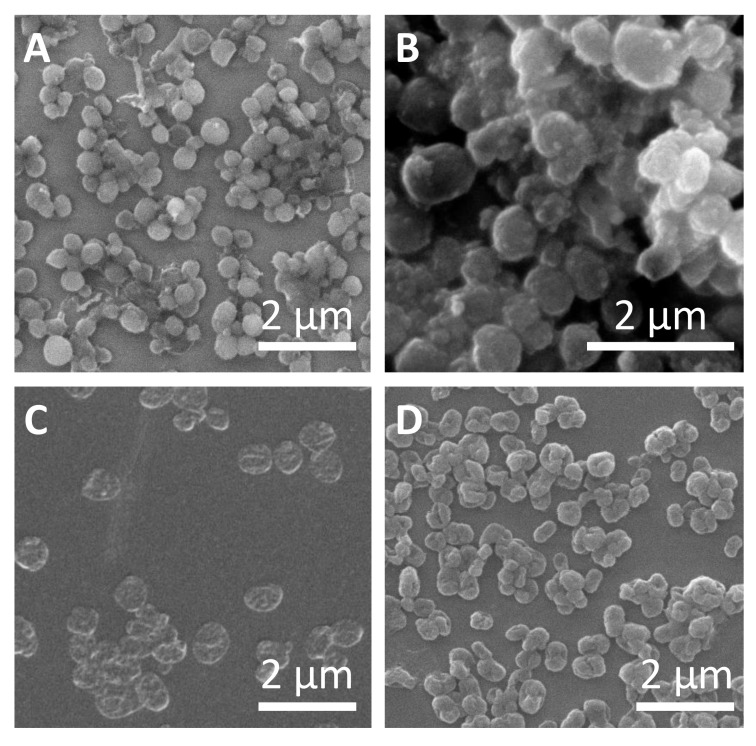
Characterization of submicrometric capsules. Representative SEM images of (**A**) SiO_2_ capsules with low amount of tetraethyl orthosilicate (TEOS), (**B**) SiO_2_ capsules with high amount of TEOS, (**C**) (DEXS/PARG)_4_ capsules, and (**D**) (PSS/PAH)_4_ capsules.

Representative micrographs of the different sub-micrometric capsules are shown in Figure 2. The average capsules size (d_c_) as derived from SEM images, hydrodynamic diameters (d_h_), and zeta potentials (ζ) of all obtained samples are presented in Table 1. Slightly higher capsule size was observed for SiO_2_ capsules synthesized with higher amounts of TEOS, which can be attributed to the thicker capsule wall. In all cases, the capsules were obtained free of aggregation, as it is shown in confocal laser scanning microscopy (CLSM) images (Figures S4–S10). Polyelectrolyte capsules (DEXS/PARG)_4_ and (PSS/PAH)_4_ also did not show significant aggregation during the different layer deposition. The evolution of the hydrodynamic diameter and the zeta-potential after the subsequent polyelectrolyte layer depositions is shown in Figures S2 and S3. All data and details observed during the different synthesis steps, including DLS and ζ-potential measurements, are provided in the Supplementary Material, as well as the different techniques used for the quantification of the cargo encapsulating efficiency. We should point out that the amount of encapsulated cargo was used as criterion for the quantification and comparison of the further experiments carried out with the capsules.

Submicrometric SiO_2_ and polyelectrolyte capsules were loaded with various cargoes, using the coprecipitation (i.e. preloading) strategy, as described in the Supplementary Material. The bioactive compounds used as molecular cargos are enlisted in Table 2. As previously commented on, for all the biological experiments performed in this work, the amount of added capsules was based on the amount of encapsulated cargo. Thus, the cytotoxicity, proliferation, uptake, and transfection experiments were carried out with capsules containing the same amount of encapsulated cargo.

In order to estimate the amount of encapsulated DEX-blue molecules within each capsule cavity, a calibration curve (fluorescence intensities versus dye concentration) was plotted (Figure S13B). This linear regression was further used to calculate the amount of encapsulated DEX-blue. To do so, capsules were previously dissolved to eliminate quenching effects. The detailed calculation procedure is described in the Supplementary Material. Calculated concentrations of encapsulated molecular cargoes are presented in Table S1.

The amount of encapsulated DNA in previously dissolved biodegradable capsules (SiO_2_, polyelectrolyte) was measured with the commercially available Quant-IT RiboGreen RNA Assay Kit. As before, a calibration curve of DNA was firstly plotted (Figure S15B), and the amount of encapsulated DNA from previously dissolved capsules was calculated based on such calibration curve, as described in the Supplementary Material. The calculated amount of DNA encapsulated in biodegradable capsules (SiO_2_, polyelectrolytes) is presented in Table S2.

### 2.2. Biological Characterization of Submicrometric SiO_2_, (PSS-PAH)_4_, (DEXS-PARG)_4_ Capsules

In order to perform a biological characterization of the synthesized capsule carriers, their cellular uptake [46,47], impact on cell viability and proliferation [11,48], their degradation [49,50], and their use for delivering encapsulated bioactive molecular cargo [15,51] were studied.

#### 2.2.1. Cell Viability Studies

**Figure 3:**
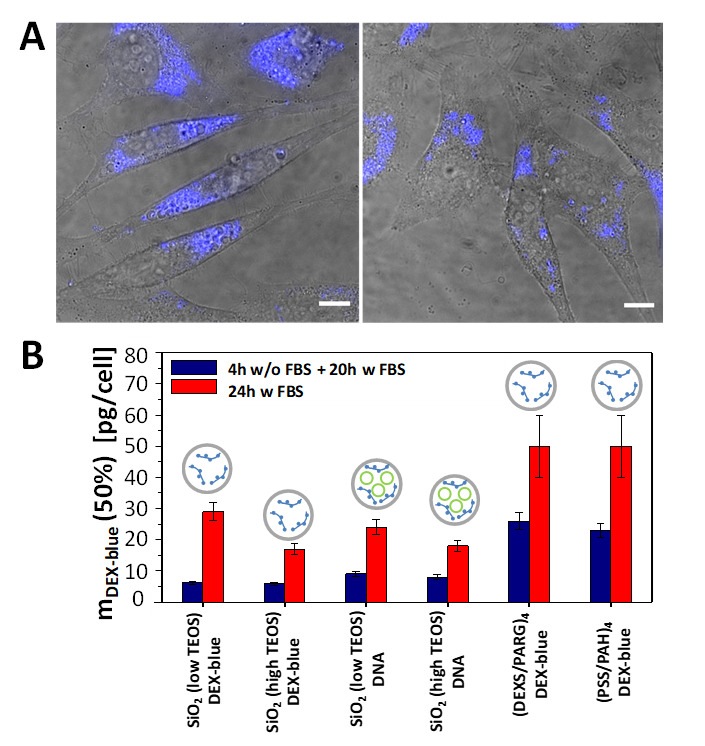
(**A**) Confocal laser scanning microscopy images (overlay of transmission and blue fluorescence channels) of HeLa cells which were exposed to SiO_2_ (low TEOS) capsules loaded with DEX-blue for 24 h in cell culture medium supplemented with FBS. The amount of added capsules was m_DEX-blue_ = 1.5 pg/cell. The scale bars correspond to 20 µm. (**B**) Cytotoxicity of submicrometric capsules: Amounts of added capsules with DEX-blue m_DEX-blue_ (50%) upon which cell viability is reduced to 50% after 24 h incubation. The following schematics was used in the above histograms: gray circles: capsules, green circles: DNA, blue structures: DEX-blue. Data are extracted from the raw data shown in Figures S16–S21.

**Figure 4:**
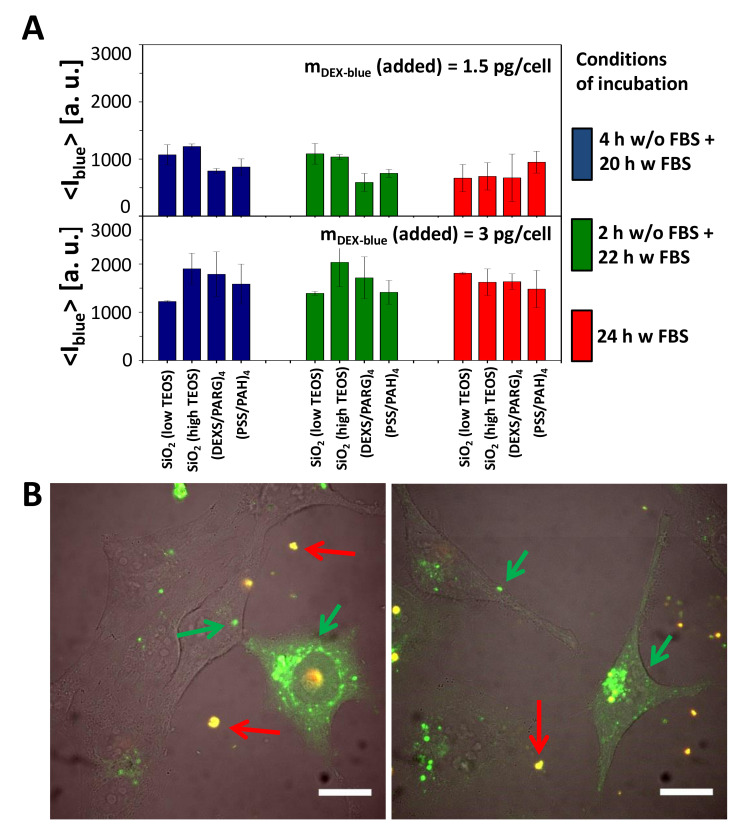
Cellular uptake of submicrometric capsules. (**A**) Mean blue fluorescence intensity <I_blue_> of HeLa cells after incubation with different submicrometric capsules loaded with DEX-blue. Cells were incubated with capsules at different conditions of incubation. The exposure concentration was m_Dex-blue_(added) = 1.5 or 3 pg/cell. About 10,000 events were analyzed. The error bars correspond to the standard deviation from 3 independent experiments. Data are taken from the raw data shown in Figures S24–S27. (**B**) Microscope image showing the uptake of SiO_2_ (low TEOS) capsules loaded with DQ-Ovalbumin (DQ-OVA) by HeLa cells. The scale bar corresponds to 20 µm. Capsules outside cells in the extracellular medium are orange (superposition of green and red), degraded capsules are green [49]. The images show also release of the encapsulated DQ-OVA to the cytosol, seen as the green fluorescent intracellular background, which is not present in all cells, and which is different from the grainy structure of green fluorescence capsules still residing in endosomes/lysosomes.

Cell viability studies were performed with the widely used fluorescence-based resazurin assay [52]. Reduction of the viability of HeLa cells upon exposure to capsules was measured using different incubation procedures: (i) first 4 h of incubation in cell growth medium without fetal bovine serum (FBS), and then 20 h of incubation in the medium supplemented with FBS; (ii) 24 h of incubation in the cell growth medium supplemented with FBS. The two conditions were used as the transfection of cells varies upon the presence/absence of FBS [53,54]. The capsule concentration was quantified in terms of delivered encapsulated DEX-blue m_DEX-blue_ [pg/cell]. Because of challenges in counting the number of capsules (due to their small size), the mass m_DEX-blue_ of encapsulated DEX-blue per capsule was hard to determine. A rough estimate based on capsule concentration determination using a hemocytometer and further calculations are presented in the Supplementary Material, Table S1. From the viability assays for the different capsules and incubation conditions the mass of delivered DEX-blue m_DEX-blue_ (50%) [pg/cell] was determined, which caused 50% reduction of cell viability (Figures S16–S21). The lower this number, the more toxic the capsules are to cells, i.e. cell viability already gets reduced at lower exposure concentrations. Capsules which had been added to cells for the first 4 h in FBS free medium had more toxic effect on cells (i.e. lower m_DEX-blue_ (50%) values) than the ones added also during the first 4 h in FBS supplemented medium. This is often attributed to the increased capsule uptake by cells when incubated for the first 4 h in FBS free medium [55], though in the present study the difference in uptake between both conditions as shown in Figure 4 for 24 h incubation time was rather non-systematic. For shorter incubation time there was some increased uptake under serum-free conditions (Figures S24–S27). Among the different types of capsules, the polyelectrolyte-based capsules showed lower toxicity, when compared with their SiO_2_-based counterparts. A possible explanation of the higher reduction in cell viability when using SiO_2_ capsules is endo/lysosomal swelling upon hydrolyzation, followed by transient poration of endosomal/lysosomal membranes [56], which is a plausible mechanism for the release of the encapsulated cargo to the cellular cytosol [20]. Focusing on the SiO_2_-based capsules, the ones synthesised with less amount of TEOS showed less reduction in cell viability, as compared to the SiO_2_ capsules with a higher amount of TEOS (Figure 3). This is in line with the hypothesis that the SiO_2_ is hydrolyzed and causes endo/lysosomal swelling, which is an indicator for toxicity [56].

Additionally, cellular proliferation during the incubation with SiO_2_ capsules was tested by measuring intracellular DNA synthesis [56]. This was directly monitored by the incorporation of the thymidine-analog EdU (5-ethynyl-2′-deoxyuridine). EdU can be detected by a copper-catalyzed click-reaction between its alkyne group and an azide group-containing the fluorescent dye [57]. According to the obtained results for HeLa cells, reduction in proliferation was caused by SiO_2_ capsules (low and high amount of TEOS) from DEX-AF647 (which was used as quantifier for capsule concentrations) concentrations higher than 3 pg/cell (Figures S28 and S29). This is in line with previous findings, that proliferation is affected already at lower particle concentrations than cell viability (i.e. the m(DEX) (50%) [pg/cell] are higher for the viability assay (Figure 3B) than for the proliferation assay, Figures S28 and S29) [56].

#### 2.2.2. Uptake Studies

The internalization of submicrometric capsules of various materials (SiO_2_ with low and high TEOS, PARG/DEXS, PAH/PSS) loaded with DEX-blue was tested with HeLa cells using flow cytometry [11,47]. It is worth noting that flow cytometry without the use of pH-sensitive fluorophores is not able to distinguish between internalized capsules and capsules attached onto the cell plasma membrane [58]. Therefore, here we are talking about capsule association with cells. Different incubation conditions were chosen to estimate the internalization (i.e., association) of submicrometric capsules by HeLa cells. Cells were incubated with the capsules (i) in the cell culture medium supplemented with FBS for 2, 4, 6, and 24 h; (ii) in the cell culture medium without FBS for 2, 4, and 6 h; (iii) first 2 h in the cell culture medium without FBS and then the medium was replaced with the cell culture medium supplemented with FBS; (iv) first 4 h in the cell culture medium without FBS and then the medium was replaced with the cell culture medium supplemented with FBS. The mean blue fluorescence signal <I_blue_> coming from each cell due to associated DEX-blue loaded capsules was then plotted versus incubation conditions (Figure 4, Figures S24–S27).

Uptake (or to be more precisely, cell-capsule association) data reveal that all types of capsules were internalized by cells at comparable amounts (Figure 4A). Addition of more capsules (m_Dex-blue_ (added) = 3 pg/cell versus 1.5 pg/cell) resulted in more internalized capsules, in agreement with previous studies [11,47]. In general there is a tendency of cells to incorporate more capsules under serum-free conditions [47,59], which often is explained by cell starvation [55,60,61]. In the present work, however, this was observed in case of short incubation times (2, 4, 6 h), but at longer incubation times (24 h) no systematic dependence in this regard was detected (Figure 4A, Figures S24–S27).

#### 2.2.3. Intracellular Degradation of SiO_2_ Capsules 

After capsule internalization by cells, they are located in endosomal/lysosomal vesicles [46,62]. However, in general, embedded molecular cargo needs to be present in the cytosol to execute its biological functionality. Endosomal/lysosomal escape is one of the key problems for particle-based delivery applications. In case of biodegradable capsules, lower pH values and enzymes that are specialized on particle digestion may induce the degradation of the capsule carriers and the subsequent release of loaded cargo [20,49]. The observation of endosomal/lysosomal swelling after NP uptake has been suggested to be connected with this carrier degradation [56], which in particular has been reported for larger silica coated capsules [20]. It is proposed that a raise in osmotic pressure then transiently perforates the endosomal/lysosomal membranes, leading to the release of the cargo to the cytosol [15,20]. This is the process as attempted with classic carriers based on cationic polymers, such as poly(ethyleneimine) (PEI). However, it is in general inefficient, as increased transient lysis of the vesicular membranes increases toxicity.

To visualize the degradation of SiO_2_-based carriers with lower and higher amounts of TEOS, DQ-Ovalbumin (DQ-OVA) saturated with BODIPY dye was loaded into the capsule cavity [49]. In its non-degraded state, due to their close proximity, the fluorescence of the BODIPY molecules is mostly self-quenched. Enzymatic degradation of DQ-OVA leads to green fluorescence of the dye through the increase of the distance between the dye molecules [49,63]. Confocal microscopy images of HeLa cells that have been incubated with both types of SiO_2_-based capsules for 24 hours (Figure 4B) showed two types of fluorescence signals. Non-internalized capsules were characterized by the superposition of red and green fluorescence signals (red fluorescence is associated with dye dimers [64], and green fluorescence originates from non-quenched dye molecules), indicated with red arrows in Figure 4B. The bright green fluorescence spots (shown with green arrows) can be ascribed to capsules located in endosomal/lysosomal vesicles, where it is known that capsules start to degrade [20] and proteases decompose DQ-OVA [49,63]. The green background fluorescence in Figure 4B of cells with internalized capsules indicates that some decomposed DQ-OVA has been released to the cytosol. Hereby transient lysis of the endosomal/lysosomal membranes surrounding the capsules has most likely occurred by the hydrolyzation of SiO_2_ [20]. As there is higher green fluorescence in the grainy spots representing DQ-OVA inside endosomes/lysosomes as compared to the green background fluorescence representing DQ-OVA in the cytosol (Figure 4B), the release of DQ-OVA is not complete and not efficient, and in addition accompanied by higher toxicity of the SiO_2_-based capsules as compared to the control capsules (Figure 3B).

#### 2.2.4. Stability of DNA in Biological Fluids

**Figure 5:**
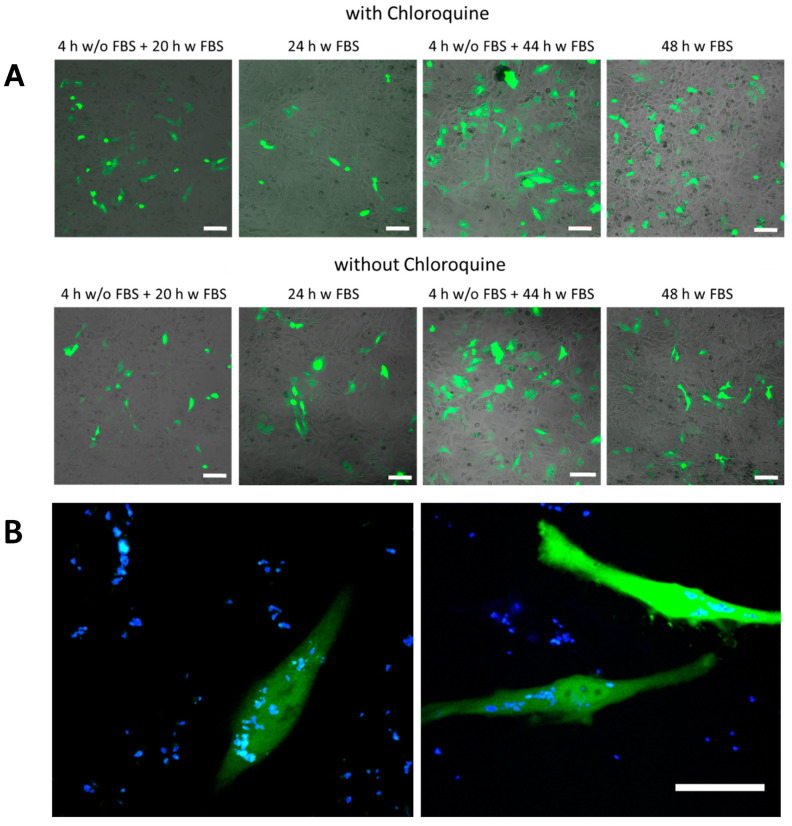
Transfection studies. (**A**) green fluorescence proteins (GFP) expression observed by confocal laser scanning microscopy of SiO_2_ (low TEOS) capsules in HeLa cells using different exposure conditions. The added amount of capsules was m_DNA_ (added) = 25 pg/cell. The scale bars correspond to 100 µm. (**B**) An example of transfected cells at higher magnification using SiO_2_ (low TEOS) capsules. The green channel corresponds to the GFP fluorescence, the blue channel to co-encapsulated DEX-blue. The scale bar corresponds to 20 µm.

**Figure 6:**
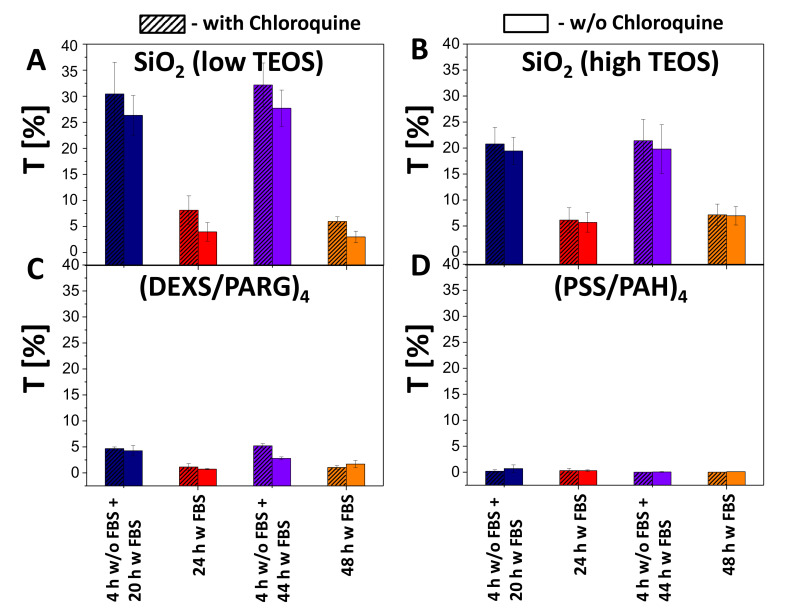
Transfection studies. Transfection efficiency T of HeLa cells by (**A**) SiO_2_ (low TEOS), (**B**) SiO_2_ (high TEOS), (**C**) (DEXS/PARG)_4_, (**D**) (PSS/PAH)_4_ capsules using different incubation conditions at 25 pg(DNA)/cell added in presence (hatched boxes) or absence (blank boxes) of chloroquine.

Apart from proteins, as demonstrated above for the case of DQ-OVA, SiO_2_-based capsules can also be used to deliver genetic material into cells [20]. As previously mentioned, after endocytosis capsules are located in endosomes/lysosomes, that contain more than 60 different enzymes for digesting alien compounds [65]. Moreover, lysosomal compartments possess acidic pH, leading to a faster degradation of its content. All these facts can affect the transfection process of cells [66]. Thus, particle-based delivery of DNA/RNA for the transfection of cells may fail in two different steps. First, inside endosomes/lysosomes the DNA/RNA may be degraded. Second, as already pointed out before, there will typically be an ineffective release of the DNA/RNA from endosomes/lysosomes to the cytosol. First, we investigated the potential degradation of DNA inside endosomes/lysosomes. In order to check the influence of pH and enzymes on DNA transfection, the commercial transfection reagent Lipofectamine 2000 was used. The stability of the plasmids encoding GFP used in this study was checked in different media, including (i) phosphate buffered saline (PBS) at pH = 4 (i.e., emulating the acidic pH inside ensomes/lysosomes), (ii) pronase (a mixture of several nonspecific endo- and exoproteases that digest proteins down to single amino acids, which are present in endosomes/lysosomes) at pH 7, (iii) pronase at pH 4, and (iv) as control PBS at pH 7. After incubation, the percentage of transfected cells was determined by flow cytometry (further and detailed information is provided in the Supplementary Material). According to the obtained data, two general observations could be drawn (Figure S33). On the one hand, higher transfection efficiencies were obtained for untreated DNA, which was also dose dependent (more added DNA gave rise to more transfected cells). On the other hand, the treatment of the DNA plasmids with pronase at acidic pH decreased the subsequent rate of transfected cells. These observations indicate that the environment of lysosomal compartments may affect the stability of the plasmids and thus the transfection efficiency. In order to improve the stability of DNA inside endosomes/lysosomes, the commercial drug chloroquine was added to the cells in order to increase the pH value inside endosomes/lysosomes [47,67], and thus, increase the DNA stability (Figure 5 and Figure 6, Figures S42–S45) [68].

#### 2.2.5. Delivery of DNA Using SiO_2_-Based Capsules

In the next step DNA plasmids encoding GFP were encapsulated as molecular cargo to be delivered into HeLa cells. SiO_2_-based capsules with low and high amount of TEOS as well as biodegradable (PARG/DEXS)_4_ and non-degradable (PAH/PSS)_4_ capsules were used as carriers. Capsules were loaded with DNA and DEX-blue, which was used for allowing to localize the capsules via fluorescence microscopy. Different incubation conditions of capsules with HeLa cells were applied for the transfection studies: (i) 4 h in medium without FBS and then 20 h in medium supplemented with FBS; (ii) 24 h in medium supplemented with FBS; (iii) 4 h in medium without FBS and then 44 h in medium supplemented with FBS; (iv) 48 h in medium supplemented with FBS. It is worth noting that capsules were added to cells at different amounts, and the mass of added DNA (m_DNA_ (added)) was used as criterion for the quantification. The roughly estimated amounts of DNA per capsule (m_DNA_) for the different types of biodegradable capsules are presented in Table S2. As previously mentioned, the bioactive compound chloroquine (7 µM) was optionally used to increase the pH inside lysosomes. Successful transfection of cells resulted in the expression of GFP, and thus, green intracellular fluorescence could be observed (Figure 5, Figures S34–S37). Transfected cells were additionally visualized with confocal microscopy, and the transfection efficiency was measured by flow cytometry in terms of GFP fluorescence intensity per cell.

According to the obtained data, the association (uptake and attachment) of capsules with cells (blue fluorescence signal due to co-encapsulated DEX-blue as measured by flow cytometry) occurred in a dose dependent manner (Figures S38–S41). As expected, almost no transfection was observed for non-degradable capsules (i.e., no green fluorescence within cells was observed), although it was possible to find single fluorescent cells. The reason for this could be the leakage of cargo from the non-degradable polyelectrolyte capsules [69]. For the all types of biodegradable capsules (SiO_2_ low TEOS, SiO_2_ high TEOS, and (DEXS/PARG)_4_), the transfection efficiency T (as defined as a percentage of cells expressing GFP) was also observed to be dose dependent: with an increase of the added amount of encapsulated DNA, transfection was also increased. SiO_2_ (low and high TEOS) capsules showed around 6–7 times higher transfection (32% of transfected cells) efficiency than biodegradable (DEXS/PARG)_4_ capsules (5% of transfected cells), as summarized in Figure 6, for an added amount of 25 pg(DNA)/cell. Further data are provided in Figures S42–S45.

The increased transfection of HeLa cells in the case of SiO_2_ capsules could be explained by the following hypothesis. To transfect cells with DNA plasmids, the delivered cargo has to reach the cell cytosol [15,20,70,71]. For this, after capsule-based delivery to endosomes/lysosomes, plasmids have to be released from these intracellular vesicles [72]. According to previous studies, the escape of cargo from the endocytic vesicles may occur upon transient rupture of these compartments. The endosomal/lysosomal membrane per se is neither permeable to the capsules nor to DNA. However, swelling of endocytic vesicles caused by osmotic pressure may lead to their disruption [15,20]. This would allow the DNA to enter the cytosol of cells, and then further into the cell nucleus, ultimately allowing for expression of the encoded protein (GFP in our study). In the case of SiO_2_ capsules, swelling and rupture of lysosomes can be attributed to the osmotic pressure established after the delivery of charged compounds and the products of the SiO_2_ capsule degradation. When considering both SiO_2_-based capsules, higher transfection efficiencies were observed for the capsules synthesized with the lower amount of TEOS, which can be possibly associated with their lower cytotoxicity. It should also be pointed out that the addition of chloroquine resulted in a rise of transfection of cells by 2–5%.

## 3. Materials and Methods

Synthesis of the capsules: Capsules were synthesized from submicrometric CaCO_3_ cores precipitated at room temperature in the presence of the desired biological cargo molecules in an ethylene glycol (EG): water mixture (5:1 in volume) [73]. After washing and centrifugation steps, the CaCO_3_ cores were first stabilized with 5 kDa CH_3_O-PEG-SH, and transferred to ethanol. A SiO_2_ layer was deposited by adding TEOS and NH_4_OH, and a final positive PARG (M_w_ = 15,000–70,000 Da) layer was added to render the capsules positively-charged. Two different TEOS amounts (nominally low and high TEOS) were used. Polyelectrolyte capsules were synthesized from the same CaCO_3_ cores by the Layer-by-Layer (LbL) strategy, consisting of the deposition of alternate negatively and positively-charged polyelectrolytes [43,74,75]. Capsules were loaded with different bioactive molecules: (i) Dextran labelled with Cascade Blue (DEX-blue), (ii) Dextran labelled with Alexa Fluor 647 (DEX-AF647), (iii) DQ-Ovalbumin (DQ-OVA), (iv) DNA plasmids encoding GFP.

Characterization: Capsules were observed by confocal laser scanning microscopy (CLSM, Meta 510, Zeiss, Jena, Germany), transmission electron microscopy (TEM, JEOL Model JEM 3010, Peabody, MA, USA) operating at an acceleration voltage of 300 keV and scanning electron microscopy (SEM-FEG Hitachi S4800, Krefeld, Germany). Particle size distributions were obtained from the CLSM micrographs by measuring the sizes of about one hundred of particles. Dynamic Light Scattering (DLS) and Zeta (ζ) potential measurements of the capsule suspensions in water were carried out at pH 7 in a Malvern Zetasizer Nano-ZS90 apparatus (WR14 1XZ United Kingdom). The amount of encapsulated biological cargo molecules was quantified by different fluorescence-based assays.

Cell culture: Human cervical carcinoma cells (HeLa) were grown in Dulbecco's Modified Eagle’s Medium (DMEM) supplemented with 10% vol. fetal bovine serum (FBS) and 1% penicillin/streptomycin at 37 °C in 5% CO_2_.

Degradation of capsules: The degradation of SiO_2_ capsules was qualitatively estimated with CLSM analysis. For this, HeLa cells were seeded into 8-well plates at an amount of 25,000 cells/well, with 250 µL of medium added per well. After 24 h, SiO_2_ capsules (low and high TEOS) loaded with DQ-OVA were added to the cells in each well (2 µL of the capsule stock solution) and HeLa cells were incubated with capsules for another 24 h. After that, confocal microscopy images were taken. Non-degraded DQ-OVA is self-quenched and emits a fluorescence signal in the red spectrum, whereas a bright green fluorescence signal is a sign of degraded DQ-OVA and, therefore, capsule degradation [63].

Uptake studies: HeLa cells were seeded into 24-well plates (500 µL of medium added per well). On the next day, capsules of each type loaded with DEX-blue were added to the cells at different amounts. HeLa cells were incubated with the different capsules at 37 °C, 5% CO_2_ at different conditions: (i) in the cell culture medium supplemented with FBS for 2, 4, 6, and 24 h; (ii) in the cell culture medium without FBS for 2, 4, and 6 h; (iii) first 2 h in the cell culture medium without FBS and then the medium was replaced with the cell culture medium supplemented with FBS; (iv) first 4 h in the cell culture medium without FBS and then the medium was replaced with the cell culture medium supplemented with FBS. After incubation, cells were washed 3 times and detached with trypsin-EDTA. Detached cells with associated capsules were dispersed in phosphate buffered saline (PBS) and the blue fluorescence signal originating from the capsules was recorded with a flow cytometer (LSRFortessa, BD, San Jose, CA, USA). Finally, the mean fluorescence intensity of the blue signal per cell was calculated and plotted versus the conditions of incubation.

Toxicity studies: HeLa cells were seeded into a 96-well plate with each well filled with V_medium_ = 100 μL and left for 24 h. After 24 h, the growth medium was replaced with growth medium containing different types of capsules at different concentrations. Cells were then incubated with capsules at following conditions: (i) first 4 h of incubation in the cell growth medium without FBS, and then 20 h of incubation in the medium supplemented with FBS; (ii) 24 h of incubation in the cell growth medium supplemented with FBS. Then, the cells were washed once with PBS, and fresh cell growth medium containing 10% vol. resazurin was added to each well for 4 h at 37 °C and 5% CO_2_. The fluorescence was measured at 560 nm excitation and 575 nm emission wavelengths, using a fluorimeter (Fluorolog-3, Horiba JOBIN YVON, Bensheim, Germany). The viability of the hMSCs was assumed to be proportional to the recorded fluorescence intensity [56].

Transfection experiments: HeLa cells were seeded into 24-well plates at an amount of 40,000 cells/well (medium per well: V_medium_ = 0.5 mL). Afterwards, capsules containing DNA plasmid and DEX-blue as molecular cargos were added to the cells at different amounts. Capsules were then incubated with cells (i) for 4 h in medium without FBS and then for 20 h in medium supplemented with FBS; (ii) for 24 h in medium supplemented with FBS; (iii) for 4 h in medium without FBS and then for 44 h in medium supplemented with FBS; (iv) for 48 h in medium supplemented with FBS. Additionally, 7 µM chloroquine was added to the cells (in cases when FBS-free medium was replaced by medium with FBS after 4 h of incubation, an additional 2 µM chloroquine was added). After incubation, cells were washed 3 times and detached with trypsin-EDTA. The blue (originating from DEX-blue) and green (originating from expressed GFP) fluorescence signals coming from each single cell were recorded with flow cytometry. Finally, the mean blue fluorescence intensity per cell, as well as the percentage of transfected cells (determined as cells with green fluorescence signal that was above the green fluorescence signal of untreated cells), was plotted versus the incubation conditions.

The complete details of all the experiments are provided in the Supplementary Material.

## 4. Conclusions

SiO_2_ capsules of submicrometric size were synthesised after the optimization of already established protocols yielding micrometric-sized capsules. The synthesis consisted of the deposition of a SiO_2_ layer onto submicrometric CaCO_3_ cores precipitated at room temperature in an ethylene glycol-water solution in presence of the desired cargo. Polymeric counterparts in the form of (PSS/PAH)_4_ and (DEXS/PARG)_4_ capsules were also synthesised for comparative reasons. The obtained capsules were shown to be suitable for the delivery of different molecules of biomedical interest, such as dextran, proteins (DQ-OVA), and also genetic material (DNA plasmids encoding GFP). Our results point out that SiO_2_-based capsules demonstrated higher efficiencies of transfection (up to 30%) compared to their polymer-based submicrometric and biodegradable counterparts (around 5%), to the costs of higher toxicity. This is the same dilemma as observed with classical PEI-based transfection. Improved endo/lysosomal escape of DNA to the cytosol goes hand in hand by increased toxicity. The increase of transfection may lie in optimizing the window of opportunity of different carriers, which may allow for improved transfection as tolerable toxicity.

## Figures and Tables

**Table 1:** Capsule diameter d_c_ as determined from SEM and hydrodynamic diameters d_h_ and Zeta-potential (ζ) as determined from dynamic light scattering (DLS) measurements.

Capsule Architecture	d_c_ [nm]	d_h_ [nm]	ζ [mV]
SiO_2_ (low TEOS)	602 ± 124	744 ± 25	29 ± 2
SiO_2_ (high TEOS)	686 ± 195	753 ± 58	27± 1
(DEXS/PARG)_4_	625 ± 71	762 ± 81	20 ± 1
(PSS/PAH)_4_	694 ± 95	690 ± 20	16 ± 1

**Table 2:** Amounts of biological molecules (volume V_bio_ and concentration C_bio_ of stock solution) added as molecular cargo for later encapsulation to the initial CaCl_2_·2H_2_O solution, V(CaCl_2_ solution) = 4 mL.

	V_bio_ [µL]	C_bio_ [mg/mL]
Dextran labelled with Cascade Blue (DEX-blue) in water	25	6.5
Dextran labelled with Alexa Fluor 647 (DEX-AF647) in water	25	6.5
DQ-Ovalbumin (DQ-OVA) in water	200	2
GFP plasmids in Tris-EDTA buffer together with	200	1.8
Dextran Cascade Blue (DEX-blue) in water	25	6.5

## Supplementary Materials

Supplementary material can be found at https://www.mdpi.com/1422-0067/21/20/7563/s1.
