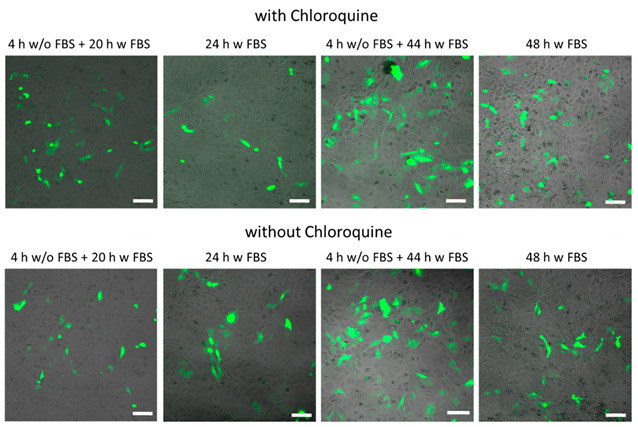# Correction: Zyuzin et al. Development of Silica-Based Biodegradable Submicrometric Carriers and Investigating Their Characteristics as in Vitro Delivery Vehicles. *Int. J. Mol. Sci.* 2020, *21*, 7563

**DOI:** 10.3390/ijms26052261

**Published:** 2025-03-04

**Authors:** Mikhail V. Zyuzin, Dingcheng Zhu, Wolfgang J. Parak, Neus Feliu, Alberto Escudero

**Affiliations:** 1 Department of Physics and Engineering, ITMO University, Lomonosova 9, St. Petersburg 191002, Russia; mikhail.zyuzin@metalab.ifmo.ru; 2 Center for Hybrid Nanostructures (CHyN), Universität Hamburg, 22607 Hamburg, Germany; dzhu@physnet.uni-hamburg.de (D.Z.); wolfgang.parak@uni-hamburg.de (W.J.P.); 3 Fraunhofer Center for Applied Nanotechnology (CAN), 20146 Hamburg, Germany; 4 Departamento de Química Inorgánica, Facultad de Química, Universidad de Sevilla, Calle Profesor García González 1, E–41012 Seville, Spain; 5 Instituto de Investigaciones Químicas (IIQ), Universidad de Sevilla–CSIC, Calle Américo Vespucio 49, E–41092 Seville, Spain

In the original publication [1], there was a mistake in Figure 5A and Figure S34 (both figures are identical). The authors apologize for this mistake and have verified that this error occurred due to the use of one image from an incorrect data series, which led to images from the same sample being used with different captioning, incorrectly described as two different experimental conditions (the left column of the original Figure 5A and Figure S34). Below the revised Figure 5A (Figure S34) is shown. All fluorescence recordings were performed in triplicate and thus in the corrected Figure 5A (Figure S34) the images in the left column are taken from a different recording. 

We note that our interpretation was not based only on individual images (Figure 5A), and that flow cytometry data were also used (Figure 6). For the low TEOS 4 h w/o FBS + 20 h FBS data, Figure 6 shows that for both conditions the transfection is similar. As commented on in the manuscript, the addition of chloroquine resulted in a slight rise in the transfection of cells (around 2–5%). However, even when the same trend is observed for these SiO_2_-based capsules (as shown in Figure 6), such numbers are within the error bars. Consequently, also in Figure 5A (Figure S34) for both conditions (with or without chloroquine) a similar fluorescence is to be expected. This can now be seen in the revised Figure 5A (Figure S34).

The authors also want to point out that, in all of the images shown in Figure 5A (Figure S34), there is an artifact in the form of a little light spot in the center of the image, which cannot be seen by the naked eye. This light spot is caused by scattering in the optical pathway of the fluorescence microscope due to alignment problems, and thus is present in all images. This, however, does not affect the interpretation of the images, as the artifact is so small that it basically cannot be seen in the images.

The authors state that the scientific conclusions are unaffected. This correction was approved by the Academic Editor. The original publication has also been updated.