# Supplementary material for: Development of Silica-Based Biodegradable Submicrometric Carriers and Investigating Their Characteristics as in Vitro Delivery Vehicles
Source: Int J Mol Sci. 2020 Oct 13;21(20):7563. doi: 10.3390/ijms21207563 (PMC7590072; doi:10.3390/ijms21207563)
Supplement: Supplementary file 1 [file ijms-21-07563-s001.zip › ijms-918940-supplementary.pdf]

Table of Contents

1. Materials
2. Synthesis of $CaCO_3$ core particles loaded with various bioactive molecules
3. Synthesis of biodegradable SiO_2 capsules
4. Synthesis of biodegradable $(DEXS/PARG)_4$ capsules
5. Synthesis of non-degradable $(PSS/PAH)_4$ capsules
6. Structural characterization of the obtained materials
7. Estimation of the amount of encapsulated cargo
8. Cell culture protocols
9. Cytotoxicity studies
10. Capsule uptake studies
11. Proliferation studies
12. Capsule degradation
13. Stability of DNA plasmids
14. In vitro transfection studies
15. References

1. Materials

Sodium carbonate (Na_2CO_3, Sigma #451614), calcium chloride dihydrate, ($CaCl_2{\cdot}2H_2O$, Sigma #223506), ethylene glycol (EG, anhydrous, 99.8%, Sigma-Aldrich #324558), DQ-Ovalbumin (DQ-OVA, M_w ~45 kDa, ThermoFischer #D12053), Dextran Alexa Fluor®647 (DEX-AF647, M_w = 10,000 Da, Thermo Fisher, #D22914), Dextran Cascade Blue (DEX-blue, M_w = 10,000 Da, Thermo Fisher, #D1976), poly(sodium 4-styrenesulfonate) (PSS, M_w = 70,000 g/mol, #243051), poly(allylamine hydrochloride) (PAH, M_w ~ 17,500 g/mol, Aldrich #283215), dextran sulfate sodium salt from *Leuconostoc spp.* (DEXS, M_w = 10,000 g/mol, Sigma-Aldrich # D4911), poly-L-arginine (PARG, M_w = 15,000-70,000 g/mol, Sigma #P7762), sodium chloride (NaCl, Sigma #S7653), α-methoxy-ω-mercapto polyethylene glycol (CH_3O-PEG-SH, M_w = 5,000 g/mol, Rapp Polymere #125000-40), ethanol (absolute, ≥ 99.8%, Sigma #24102), ammonium hydroxide solution (NH_4OH, Sigma #338818), tetraethylorthosilicate (TEOS, 98%, Sigma #131903), and ethylenediaminetetraacetic acid (EDTA, ThermoFisher #15575020) were used for the preparation of the capsules. Milli-Q water with a resistance of 18.2 MΩ cm^{-1} was employed for all experiments.

2. Synthesis of $CaCO_3$ core particles loaded with various bioactive molecules

Submicrometer sized capsules were prepared by modified well-established methods [1,2]. Sub-micrometer sized $CaCO_3$ cores were produced by precipitation of $CaCl_2{\cdot}2H_2O$ and Na_2CO_3 solutions in an ethylene glycol: water mixture (5:1 in volume) in the presence of the desired cargo molecules [3,4]. For each batch, 4 mL of $CaCl_2{\cdot}2H_2O$ (0.33 M in a 5:1 (v/v) ethylene glycol (EG):water solution) were mixed with the different cargo molecules (DNA plasmid encoding green fluorescence protein (GFP), DQ-Ovalbumin (DQ-OVA), and dextran labeled with Cascade Blue (DEX-blue) or Alexa Fluor 647 (DEX-AF647), as indicated in **Table 2**) under magnetic stirring at 1000 rpm.

Afterwards, 772 μL of Na_2CO_3 (0.33 M in a 5:1 EG: water solution) were added to the mixture and left under stirring for 20 min at room temperature. The formed $CaCO_3$ cores were then transferred into Eppendorf tubes (2 mL), centrifuged at 8,000 rpm, washed twice with ethanol (i.e. adding around 2 mL of ethanol, sonicating them for around 30 s until particle redispersion, centrifuging at 8,000 rpm, and removing supernatants), and one time with MilliQ water. The $CaCO_3$ particles containing the cargo were finally dispersed in MilliQ water. Each core batch was divided into four portions, which were used as templates to prepare four different types of sub-micrometer sized capsules: SiO_2 (namely, low and high TEOS), $(DEXS/PARG)_4$ and $(PSS/PAH)_4$, which is described in the following sections.

3. Synthesis of biodegradable SiO_2 capsules

A silica (SiO_2) shell was grown around the $CaCO_3$ core particles as follows: one portion of the $CaCO_3$ core suspension was centrifuged at 8,000 rpm for 3 min, and then the supernatant was removed. Afterwards, 1.5 mL of a 5 kDa CH_3O-PEG-SH solution at a concentration of 6 mg/mL were added to stabilize the

cores, and the suspension was sonicated for 30 min at room temperature [2]. The cores were then washed one time with MilliQ water, one time with ethanol (i.e. adding around 2 mL of the solvent, centrifuging at 8,000 rpm, and removing the supernatant), and were finally dispersed in 2 mL of ethanol. The cores were then sonicated for 5 min and added to a glass beaker containing 16.1 mL of ethanol, 4 mL of MilliQ water, 230 μL of commercial NH_4OH, and 60/100 μL of TEOS (for low/high TEOS amount). The mixture was stirred at 500 rpm for 3 h. After coating, the particles were washed one time with ethanol and one time with MilliQ water. To dissolve the inorganic $CaCO_3$ cores, 0.5 mL of 0.2 M EDTA at pH 6 were added, and the sample was kept overnight at 4 ºC. After core dissolution, the empty capsules were washed twice with MilliQ water and centrifuged at 1500 rpm for 45 min. A final positive PARG layer was then deposited onto the capsule surface by adding 500 μL of an aqueous solution of PARG (5 mg/mL, pH 6.5, 0.05 M in NaCl). The mixture was sonicated for 3 s, shaken for 15 min, and finally washed again twice with MilliQ water, as described above. The capsules were finally dispersed in 500 μL of MilliQ water.

4. Synthesis of biodegradable $(DEXS/PARG)_4$ capsules

Biodegradable $(DEXS/PARG)_4$ capsules were prepared following the conventional Layer-by-Layer (LbL) strategy, but with higher concentrated polyelectrolyte solutions, reduced amount of NaCl, and lower molecular weight polyelectrolytes than the ones usually used for the synthesis of micrometer sized capsules [5]. This was employed to minimize aggregation during the different coating processes [6]. For the synthesis of the $(DEXS/PARG)_4$ capsules, one portion of the $CaCO_3$ core suspension was centrifuged at 8,000 rpm and the supernatant was removed. Afterwards, 0.5 mL of a DEXS solution (10 mg/mL, pH 6.5, prepared in 0.05 M of NaCl) were added to the cores. The dispersion was sonicated for 5 min and then shaken for 10 min. The excess of polyelectrolyte was removed by 2 washing steps with 2 mL of MilliQ water via centrifugation at 6,000 rpm for 3 min. Afterwards, 0.5 mL of a PARG solution (5 mg/mL, pH 6.5, prepared in 0.05 NaCl) were added to the cores. The dispersion was then sonicated for 5 min and shaken for 10 min. The cores were again washed twice with MilliQ water. The deposition process was performed 8 times, resulting in positively charged capsule shells with an $(DEXS/PARG)_4$ architecture. The $CaCO_3$ cores were finally dissolved by overnight treatment with 0.5 mL of EDTA (0.2 M at pH 6.5) at 4 ºC. Capsules were then washed twice with MilliQ water via centrifugation at 1500 rpm for 45 min, and were finally dispersed in 500 μL of MilliQ water.

5. Synthesis of non-degradable $(PSS/PAH)_4$ capsules

Non-degradable $(PSS/PAH)_4$ capsules were synthesized as described in §4 of the **Supporting Information**, but by using synthetic PAH and PSS as polyelectrolytes. The polyelectrolyte solutions were prepared in 0.05 M NaCl at pH 6.5 with a polyelectrolyte concentration of 10 mg/mL, leading to positively charged capsules with a $(PSS/PAH)_4$ architecture. The dissolution of $CaCO_3$ cores was also made with EDTA (0.5 mL, 0.2 M, pH 6), leaving the sample overnight at 4 ºC. Capsules were then washed

twice with MilliQ water via centrifugation at 1500 rpm for 45 min and discarding of the supernatant, and were finally dispersed in 500 µL of MilliQ water.

6. Structural characterization of the obtained materials

The whole synthesis process was monitored with Dynamic Light Scattering (DLS) and Laser Doppler Anemometry (LDA) using a commercial setup (Zetasizer Nano ZS, Red badge, ZEN3600, Malvern; 173° backscatter settings, 633 nm laser) for all types of capsules loaded with DEX-blue. As it can be seen in **Figure S1**, no aggregation was observed after the different synthesis steps, and the SiO_2 capsules showed a negative zeta-potential until the final coating with PARG. The evolution of the hydrodynamic diameter d_h and the zeta-potential ζ is illustrated for SiO_2 capsules with low amount of TEOS and encapsulated DEX-blue.

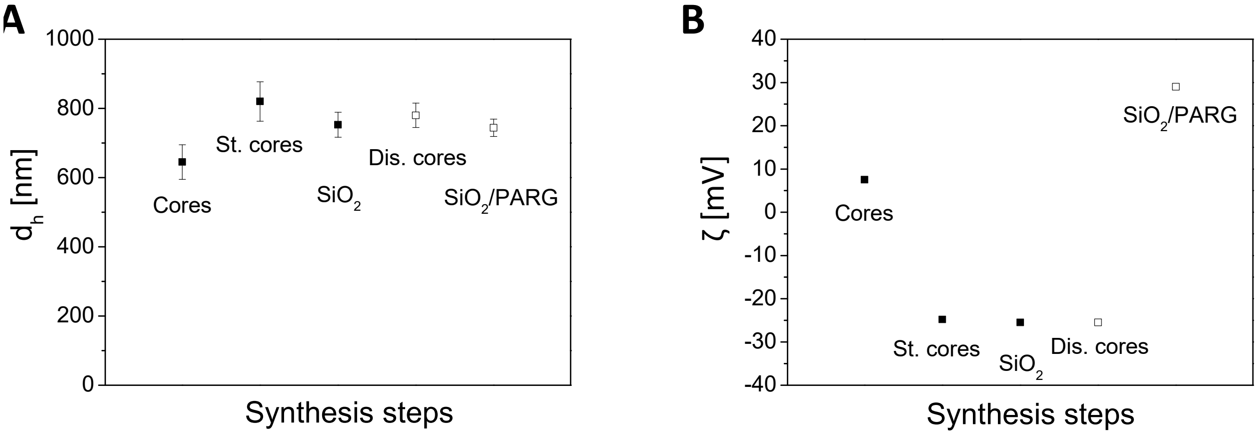

Figure S1. Evolution of A. the hydrodynamic diameter d_h and B. the zeta–potential ζ (measured at pH = 7) for SiO_2-based capsules (low TEOS) after the different synthesis steps. Empty symbols correspond to the dissolved capsules. The error bars represent the standard deviation values from three independent measurements. For the zeta-potential the error bars are smaller than the size of the symbols. "St. cores" are $CaCO_3$ cores stabilized with 5 kDa CH_3O-PEG-SH. "Dis. cores" are SiO_2 shells after dissolution of $CaCO_3$. SiO_2-based submicrometric capsules loaded with other cargos showed a similar behavior.

Polyelectrolyte capsules $(DEXS/PARG)_4$ and $(PSS/PAH)_4$ also did not show significant aggregation during the different layer deposition. The evolution of the hydrodynamic diameter and the zeta-potential after the subsequent polyelectrolyte layer deposition are shown in **Figures S2** and **S3**, illustrated again for DEX-blue-loaded capsules. In general terms, solutions with lower polyelectrolyte (2 mg/mL), and higher NaCl (0.5 M) concentrations, which are normally used for synthesis of micrometric polyelectrolyte capsules, gave rise to aggregated particles [5]. With our reported polyelectrolyte solutions (10 mg/mL, NaCl = 0.05 M for PSS, PAH DEXS, and 5 mg/mL, NaCl = 0.05 M for PARG) only minor increase of hydrodynamic diameter was observed for the non-biodegradable submicrometric capsules after the first

and second PAH deposition, whereas no major aggregation was observed for the biodegradable capsules. Capsules loaded with other cargos showed a similar behavior.

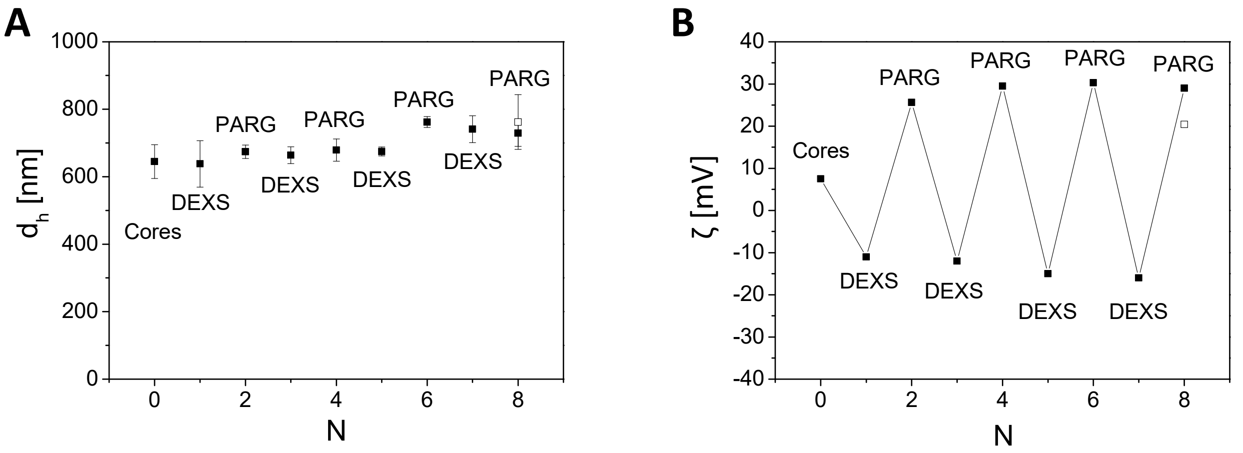

Figure S2. Evolution of A. the hydrodynamic diameter d_h and B. the zeta-potential ζ (measured at pH = 7) of the biodegradable (DEXS/PARG)$_4$ sub-micrometric capsules after the polyelectrolyte deposition steps. Empty symbols correspond to dissolved capsules. DEXS = Dextran sulfate sodium salt of 6.5 – 10 kDa; PARG = Poly-L-arginine hydrochloride of 15-70 kDa. The error bars represent the standard deviation values from three independent measurements. In case of the zeta-potential measurements the error bars are smaller than the size of the symbols).

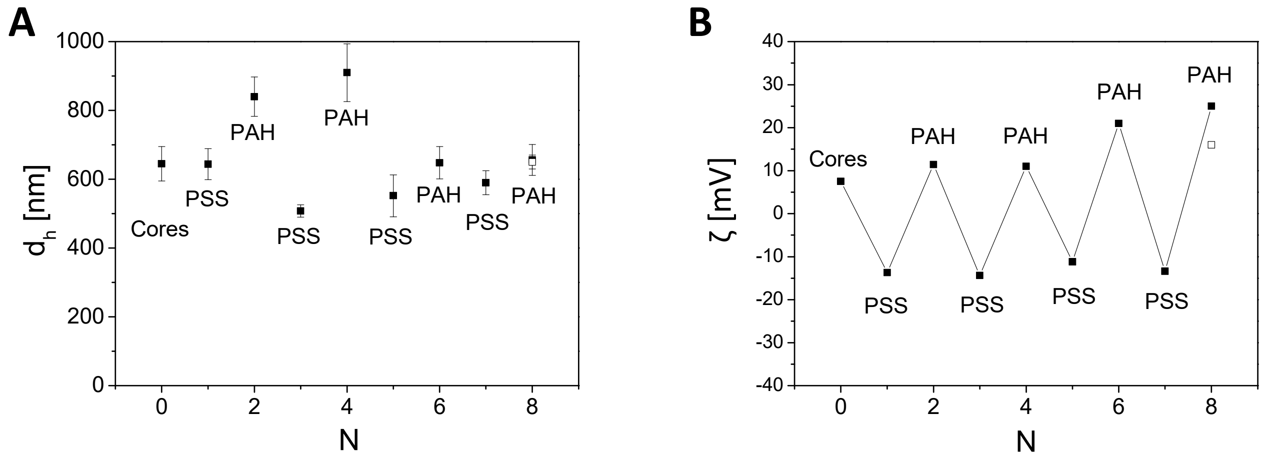

Figure S3. Evolution of (A) the hydrodynamic diameter d_h and (B) the zeta-potential ζ (measured at pH = 7), of the non-biodegradable (PSS/PAH)$_4$ sub micrometric capsules after the polyelectrolyte deposition steps. Empty symbols correspond to dissolved capsules. PAH = Poly(allylamine hydrochloride) of 17.5 kDa; PSS = Poly(sodium 4-styrenesulfonate) of 70 kDa. The error bars represent the standard deviation values from three independent measurements. In the case of the zeta-potential data the error bars are smaller than the size of the symbols.

The obtained submicrometric capsules loaded with DEX-blue, DEX-AF647 and DQ-OVA were additionally visualized with confocal laser scanning microscopy CLSM (Zeiss, Meta 510) **(Figures S4 to S10**).

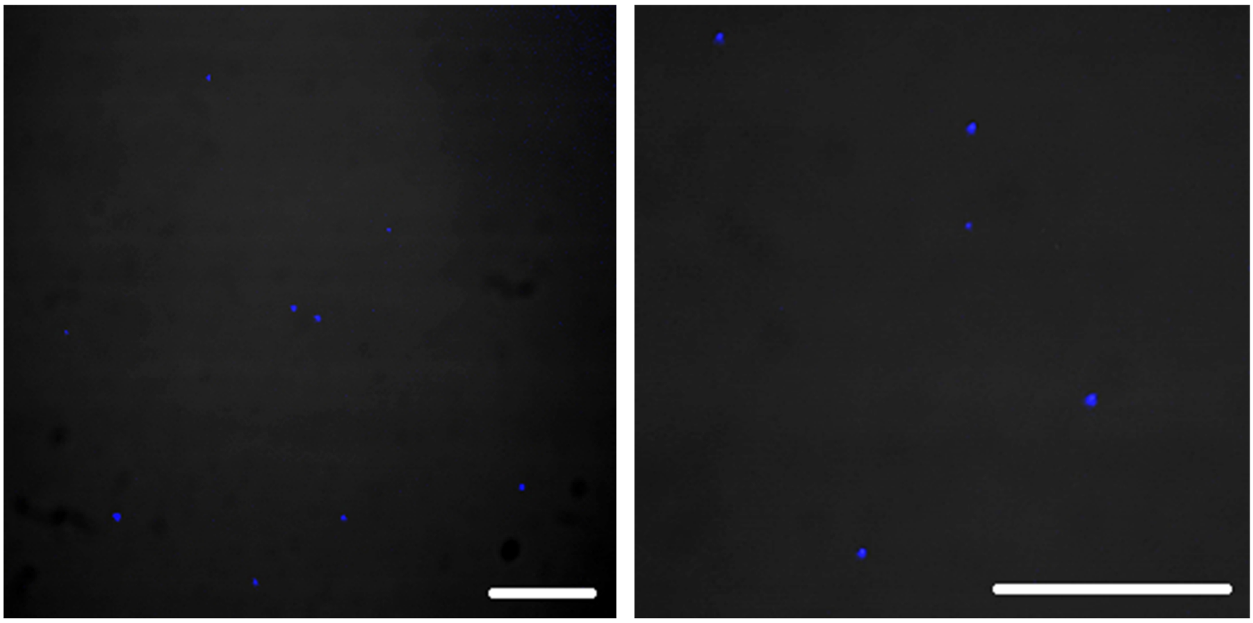

Figure S4. Representative confocal laser scanning microscopy (CLSM) images of SiO_2/PARG (low TEOS) capsules labeled with DEX-blue (excitation at 405 nm and emission with a long pass (LP) of 420 nm). Capsules were dispersed in water. The scale bars correspond to 20 μm.

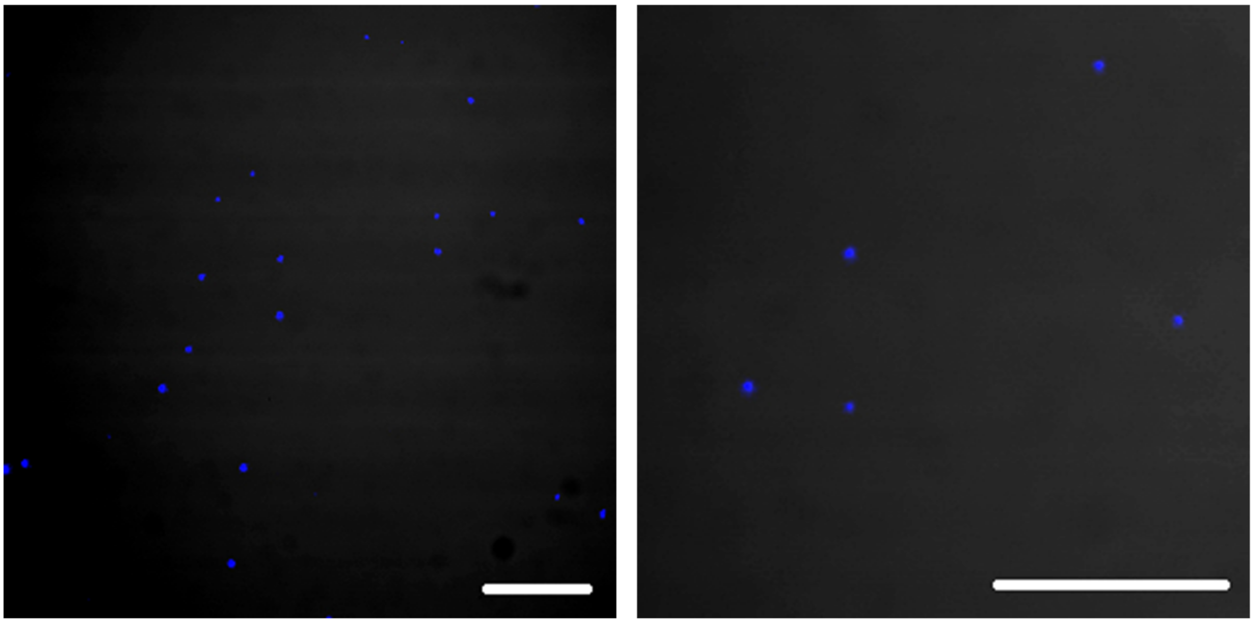

Figure S5. Representative confocal laser scanning microscopy (CLSM) images of SiO_2/PARG (high TEOS) capsules labeled with DEX-blue (excitation at 405 nm and emission with LP 420 nm). Capsules were dispersed in water. The scale bars correspond to 20 μm.

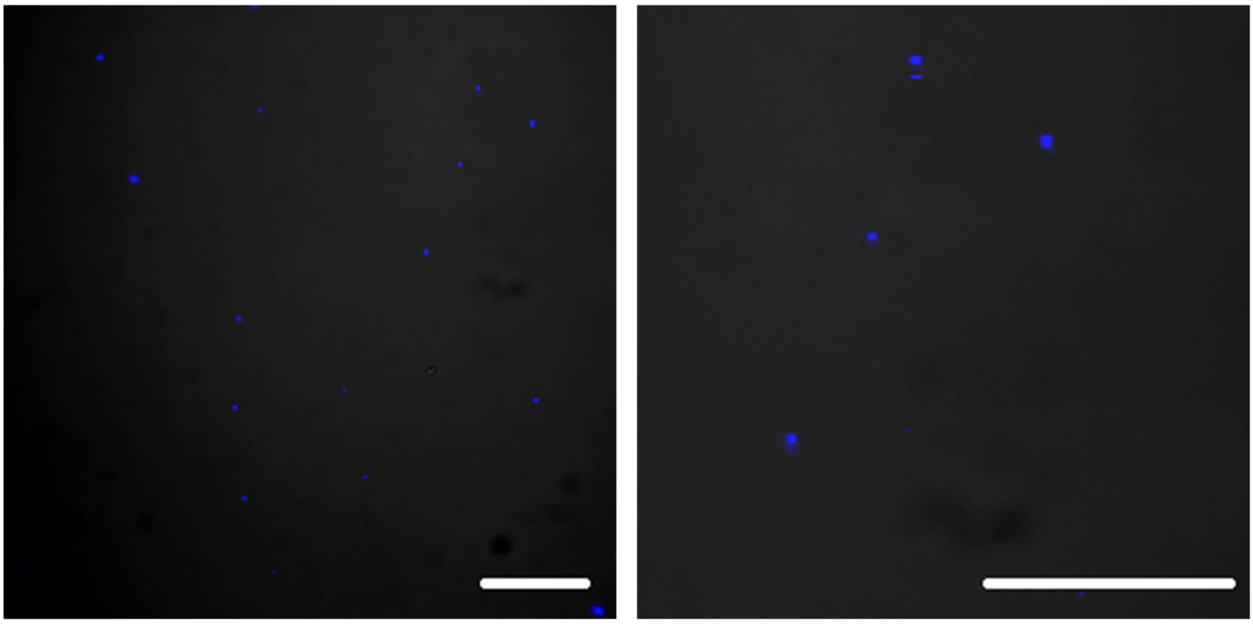

Figure S6. Representative confocal laser scanning microscopy (CLSM) images of $(DEXS/PARG)_4$ capsules labeled with DEX-blue (excitation at 405 nm and emission with LP 420 nm). Capsules were dispersed in water. The scale bars correspond to 20 μm.

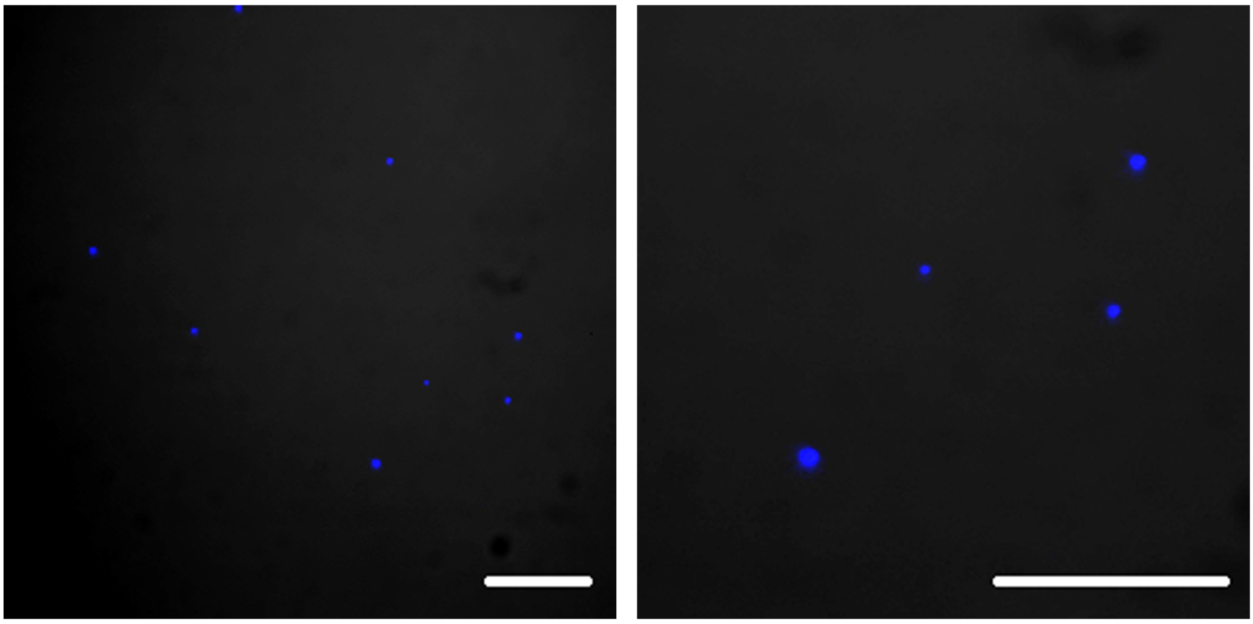

Figure S7. Representative confocal laser scanning microscopy (CLSM) images of $(PSS/PAH)_4$ capsules labeled with DEX-blue (excitation at 405 nm and emission with LP 420 nm). Capsules were dispersed in water. The scale bars correspond to 20 μm.

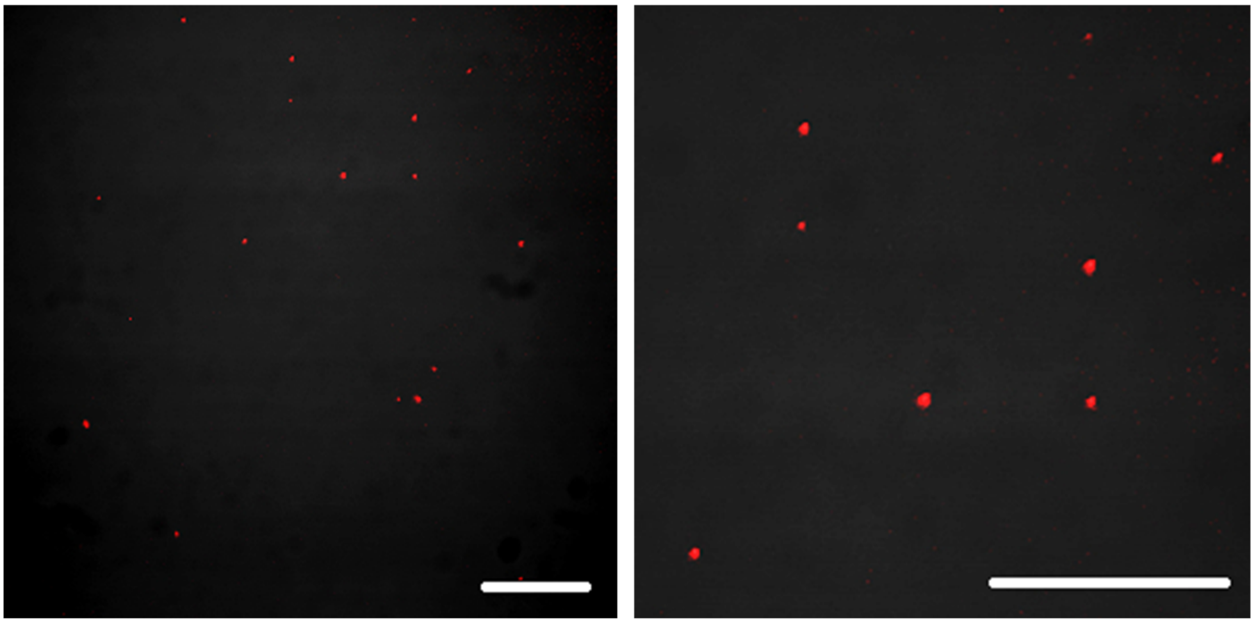

Figure S8. Representative confocal laser scanning microscopy (CLSM) images of SiO_2/PARG (low TEOS) capsules labeled with Dextran Alexa Fluor 647 (excitation at 633 nm and emission with LP 640 nm). Capsules were dispersed in water. The scale bars correspond to 20 μm.

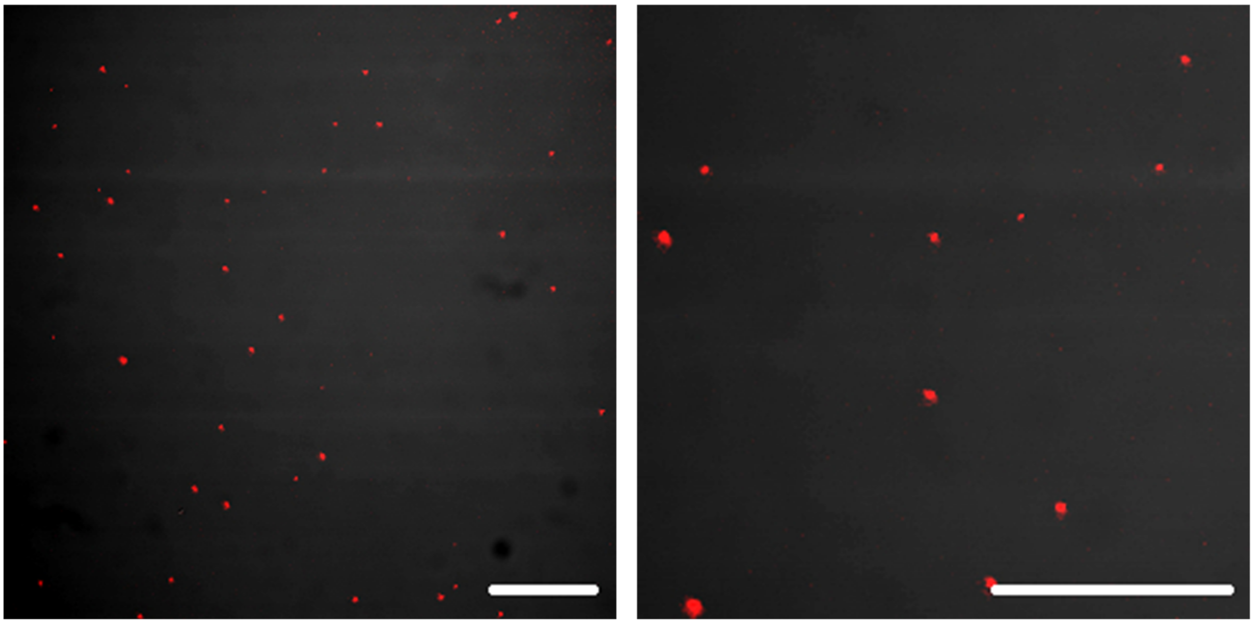

Figure S9. Representative confocal laser scanning microscopy (CLSM) images of SiO_2/PARG (high TEOS) capsules labeled with Dextran Alexa Fluor 647 (excitation at 633 nm and emission with LP 640 nm). Capsules were dispersed in water. The scale bars correspond to 20 μm.

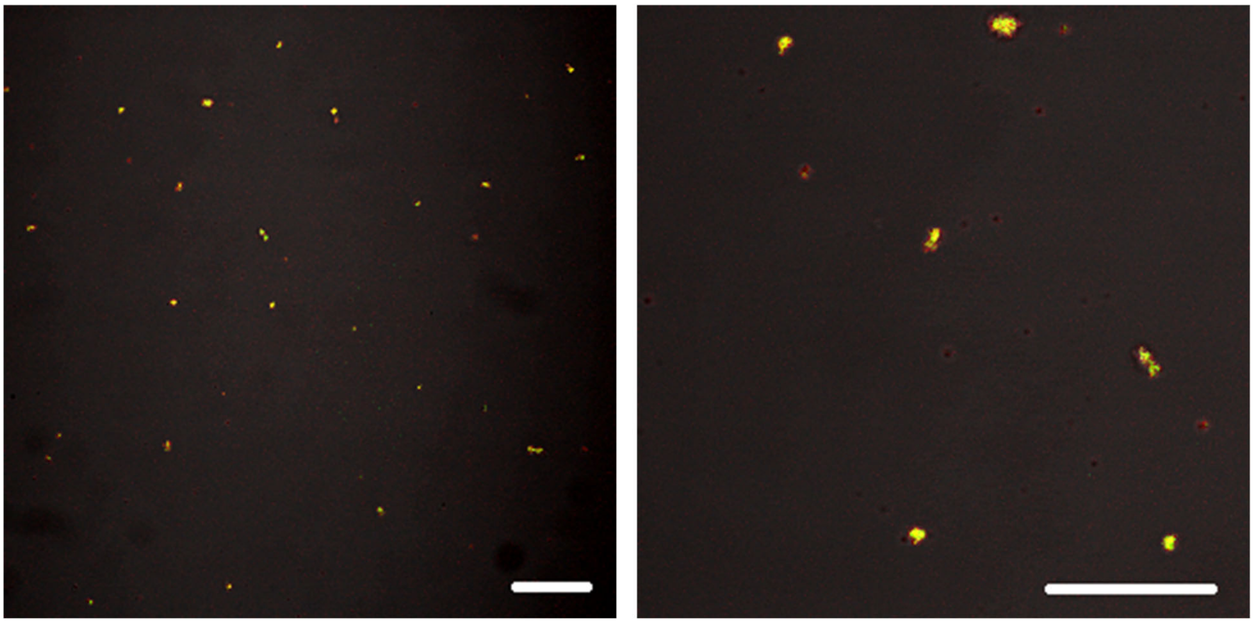

Figure S10. Representative confocal laser scanning microscopy (CLSM) images of SiO_2/PARG (low TEOS) capsules loaded with DQ-Ovalbumin (excitation at 488 nm, emission with band pass (BP) 505-580 nm and LP 620). The green fluorescence signal is coming from the not fully quenched DQ-OVA, red fluorescence signal is coming from the dye dimmers [7,8] Both signals are colocalized within one non-degraded capsule. Capsules were dispersed in water. The scale bars correspond to 20 µm.

Scanning electron microscopy (SEM) was carried out on an Inspect SEM (FEI, USA) with an acceleration voltage of 20 kV. The day before measurements, 35 µL of submicrometric capsules dispersed in water were dropped onto a glass cover slip and let dry overnight. The next day, the dry samples were coated with a thin film of gold and imaged with SEM.

Capsules were also analyzed with transmission electron microscopy (TEM Philips 200CM, SEM-FEG Hitachi S4800). Images of individual capsules are shown in **Figure S11**. Capsule diameters d_c as obtained from the microscopy data as well as the final DLS and zeta-potential data of all capsules are given in **Table 1**.

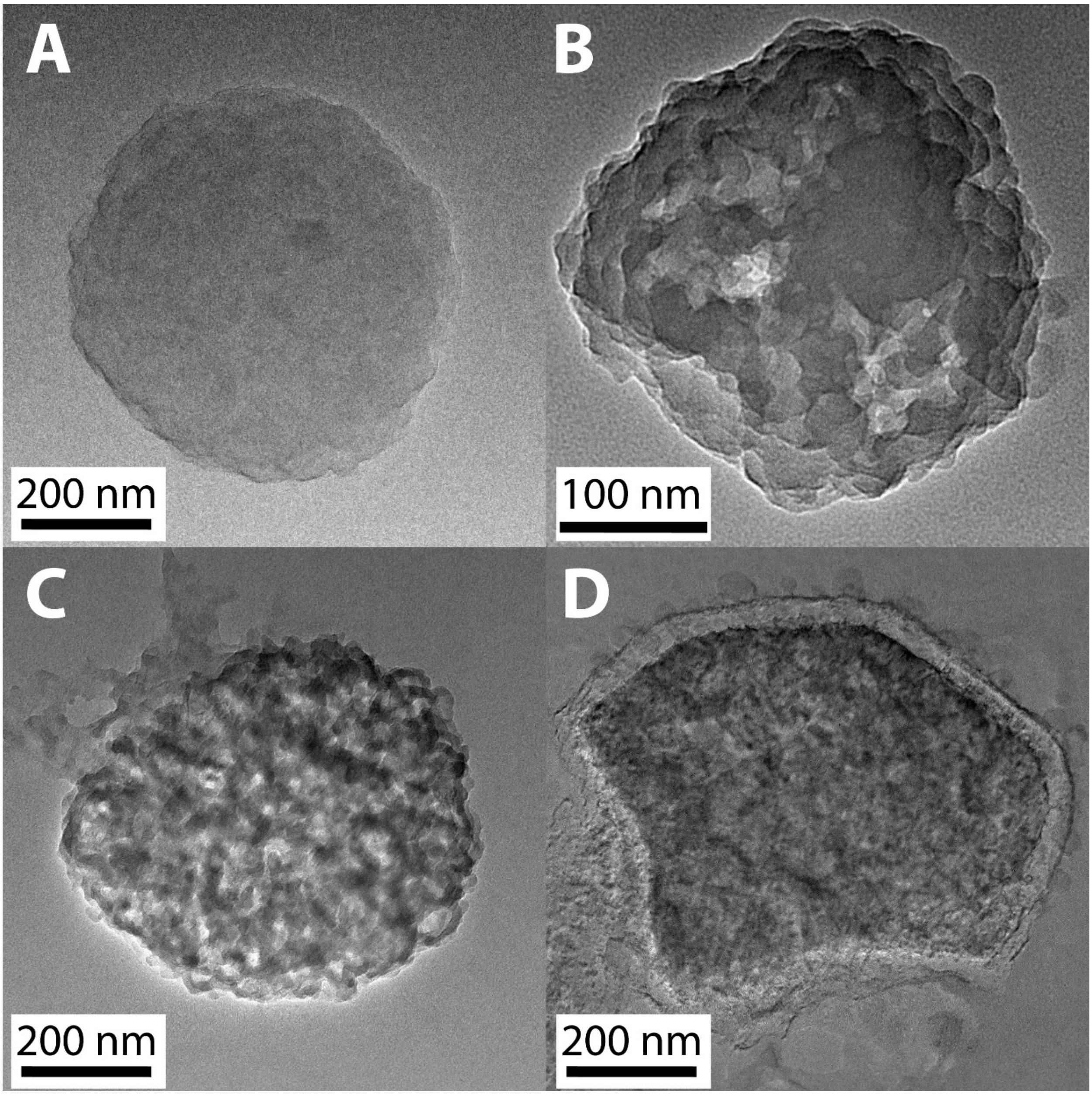

Figure S11. Transmission electron microscopy (TEM) images of submicrometric capsules. A. $(PSS/PAH)_4$, B. $(DEXS/PARG)_4$, C. SiO_2 (low TEOS), and D. broken SiO_2 capsule (low TEOS).

7. Estimation of the concentration of encapsulated cargo

Given the small size of the obtained capsules we were not able to reliably determine their concentration with a Neubauer counting chamber. Thus, capsule doses were quantified by the amount of encapsulated cargo. Therefore, uptake, cytotoxicity, proliferation, and transfection experiments were carried out with capsules containing the same amount of encapsulated molecular cargo.

To quantify the cargo amount inside each type of capsules, 250 μL of a 0.01 M NaOH solution was added to 50 μL of the polyelectrolyte-based $(DEXS/PARG)_4$ or $(PSS/PAH)_4$ capsule stock solution to promote capsule dissolution. SiO_2 capsules (50 μL of the stock solutions) were dissolved by adding 250 μL solution

of pronase (2 mg/mL, PRONASE® Protease, Streptomyces griseus, Merck, #53702). The capsule/pronase mixture (5/1 in volume) was kept overnight at 37 °C, 5% CO_2.

<u>*Estimation of the amount of DEX-blue inside capsules:*</u> The amount of encapsulated DEX-blue was estimated by fluorescence measurements. To avoid possible interference of pronase, the background spectra of DEX-blue and pronase were first collected. For this, a DEXS-blue solution with a stock concentration of 6.5 mg/mL was prepared, and it was further diluted up to $C_{DEX\text{-}blue}$ = 0.1 mg/mL, with a final volume V = 100 μL, as follows: (i) DEX-blue (1.5 μL, 6.5 mg/mL) was diluted in water (98.5 μL), (ii) DEX-blue (1.5 μL) was diluted in pronase (98.5 μL, 2 mg/mL), and (iii) water (1.5 μL) was mixed with pronase (98.5 μL, 2 mg/mL). Afterwards, the fluorescence spectra of all three samples were recorded with a fluorimeter (Fluorolog-3, Horiba JOBIN YVON) using the same conditions (excitation/emission wavelength $\lambda_{exc}/\lambda_{em}$ = 400/420 nm, respectively) at the same day. The resulting spectra are shown in **Figure S12**.

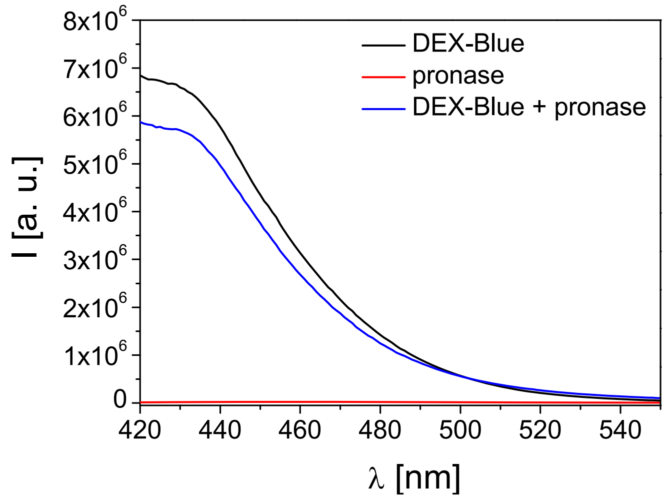

Figure S12. Fluorescence spectra I(λ) of DEX-blue mixed with pronase. Pronase and DEX-blue are the controls λ_{exc}=400 nm.

No fluorescence signal was detected from the pronase sample. The DEX-blue + pronase sample showed slightly lower fluorescence signal than the DEX-blue sample. These data suggest that pronase does not give additional signal and, thus, it is reasonable to use this fluorescence method to quantify the amount of encapsulated DEX-blue.

To determine the encapsulated DEX-blue concentration, first a calibration curve of DEX-Blue was determined. For this, the fluorescence signal of DEX-blue solutions was recorded at different dye concentrations (from $C_{DEX\text{-}blue}$ = 0 to 100 μg/mL). The fluorescence intensity I_{420} of all dilutions at λ_{exc} = 400 nm was then plotted versus the dye concentration $C_{DEX\text{-}blue}$ (**Figure S13**).

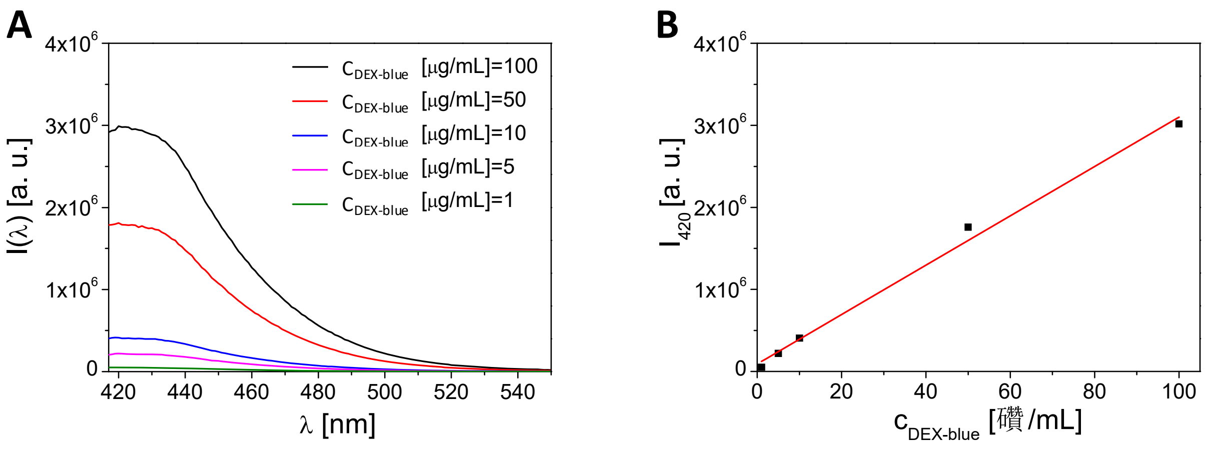

Figure S13. A. Fluorescence spectra I(λ) for different DEX-blue concentrations $C_{DEX\text{-}blue}$ with the excitation wavelength λ_{exc}= 400 nm. B. Fluorescence maximum I_{420} at λ_{em} = 420 nm (as determined from the I(λ) spectra from A) plotted versus the DEX-blue concentrations $C_{DEX\text{-}blue}$.

The calibration curve shown in **Figure S13B** was fitted with the following equation by linear regression analysis:

$$I_{420}(C_{DEX\text{-}blue}) = 30064 \text{ mL/}\mu\text{g} \cdot C_{DEX\text{-}blue} + 92945, \text{ leading to}$$

$$C_{DEX\text{-}blue}(I_{420}) = (I_{420} - 92945)/(30064 \text{ mL/}\mu\text{g})$$

The DEX-blue concentrations of the previously dissolved capsules were determined with this calibration curve. A dilution factor (f_{DF}) of 6 was taken into account (50 μL of capsule solution + 250 μL of pronase/NaOH solution, as described above). The fluorescence spectra from the solutions containing the dissolved capsules were measured, and the concentrations of the encapsulated DEX-blue $C_{DEX\text{-}blue}$ were calculated. To illustrate this, the following calculation is shown for (DEXS/PARG)$_4$ capsules. The maximum fluorescence peak at 420 nm for (DEXS/PARG)$_4$ capsules was I_{420} = 271880 a.u. From the equation of the fitted curve (**Figure S13B**) then the DEX-blue concentration from the capsule stock solution was obtained as follows:

$$C_{DEX\text{-}blue}(I_{420} = 271880) = (I_{420} - 92945)\cdot f_{DL}/(30064 \text{ mL/}\mu\text{g}) =$$

$$(271880 - 92945)\cdot 6/(30064 \text{ mL/}\mu\text{g}) = 35.7 \text{ ng/}\mu\text{L}$$

Despite the small size of the capsules to be clearly observed with the naked eye, the approximate number of submicrometric capsules per mL was roughly estimated for (DEXS/PARG)$_4$ capsules loaded with DEX-blue, using a hemocytometer (Neubauer chamber). As stated above, this value is affected by significant error bars. This resulted in $C_{capsules} = 2 \cdot 10^8$ [caps/mL]. Given that the same synthesis protocols were used,

the assumption that such figures are similar for all types of capsules was considered a good approximation. In **Table S1** the amount of encapsulated DEX-blue in the stock solutions of the 4 different types of capsules is summarized.

Table S1. Measured concentrations of loaded DEX-blue for the stock solutions of all types of synthesized capsules, and estimation of the amount of the loaded cargo per capsule.

Capsule architecture	$C_{DEX\text{-}blue}$ [ng/µL]	$C_{capsules}$ [caps/mL]	$m_{DEX\text{-}blue}$ [g/caps]
SiO_2 (low TEOS)	47.1	$2 \cdot 10^8$	$23.5 \cdot 10^{-14}$
SiO_2 (high TEOS)	44.8	$2 \cdot 10^8$	$22.4 \cdot 10^{-14}$
$(DEXS/PARG)_4$	35.7	$2 \cdot 10^8$	$17.8 \cdot 10^{-14}$
$(PSS/PAH)_4$	23.5	$2 \cdot 10^8$	$11.7 \cdot 10^{-14}$

Estimation of the amount of plasmid GFP inside capsules: The amount of encapsulated DNA (*i.e.* plasmid encoding GFP) was only estimated for the biodegradable capsules, *i.e.* for SiO_2 (low and high TEOS) and $(DEXS/PARG)_4$. Biodegradable capsules were dissolved with pronase, as described above. Since the non-biodegradable capsules could not be dissolved with pronase, and NaOH would induce the degradation of DNA, it was not possible to determine the amount of loaded DNA in $(PSS/PAH)_4$ capsules. It should, however, be mentioned that such capsules are not expected to degrade inside cells followed by release of their cargo, and were thus used as control.

The amount of encapsulated DNA could be evaluated with two methods: by measuring the absorbance at 260 nm and with the commercially available Quant-IT RiboGreen RNA Assay Kit (ThermoFisher, #R11490). Pronase has a strong absorbance at 260 nm, and it can thus interfere with the estimation of the DNA concentration. For this reason, we used a second method for the DNA quantification.

This assay kit is based on the fluorescence of a dye that links to the DNA. The concentration of DNA can thus be quantified with a fluorimeter. The possible interference of pronase and DNA was also taken into account. For this, DNA was diluted in pronase (2 mg/mL) up to a concentration of 50 µg/mL. This DNA-pronase solution was kept for 24 hours at 37 °C, 5% CO_2 in an incubator. The amount of DNA was then quantified with the Quant-IT RiboGreen RNA Assay Kit, according to the protocol of the product, which will be described later. As controls, only pronase and only DNA at the same concentrations were used (**Figure S14**).

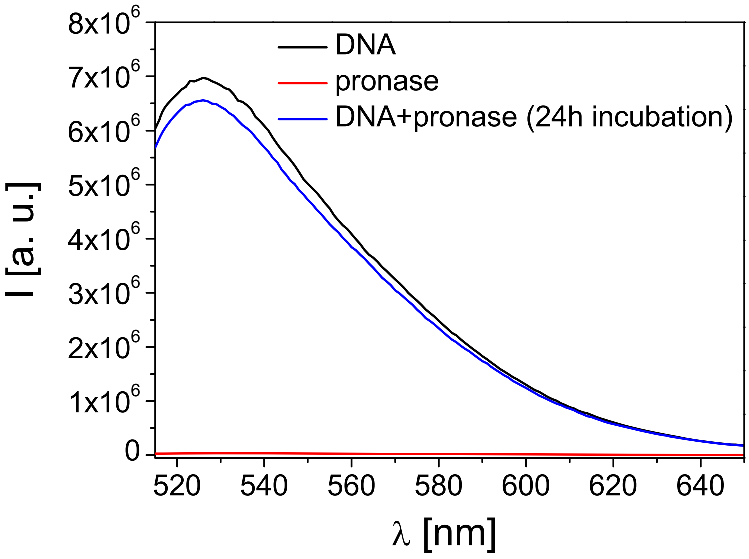

Figure S14. Fluorescence spectra I(λ) of DNA, pronase, and DNA with pronase. All compounds were previously mixed with the RNA Reagent (1:1), λ_{exc}= 480 nm.

According to the obtained results, it can be observed that pronase did not give any significant fluorescence signal, and the fluorescence signal from DNA without pronase and from DNA after 24 h incubation with pronase remained approximately at the same level. Thus, this kit was chosen to estimate the amount of encapsulated DNA.

The estimation of DNA was performed according to the manual. Briefly, the RNA reagent (included to the kit) was diluted 200 times in Tris-EDTA (TE) Buffer 1x. The stock solution of DNA was also diluted in TE 1x buffer at different known concentrations (C_{DNA} = 0-1000 ng/mL) in order to get the calibration curve of DNA. The fluorescence spectra of these samples were measured with a fluorimeter (wavelengths of excitation and emission λ_{exc} = 480 nm and λ_{em} = 530 nm, respectively), and a calibration curve was then obtained (**Figure S15**).

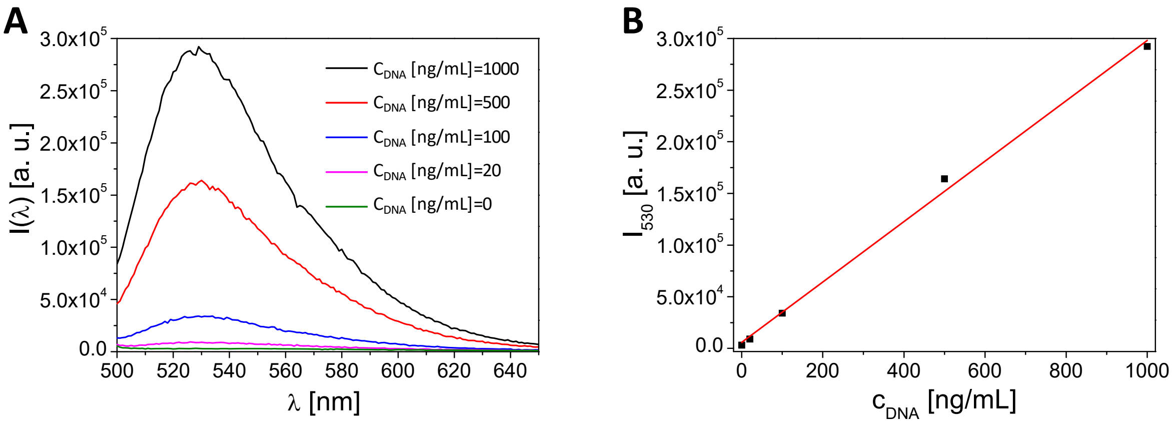

Figure S15. A. Fluorescence spectra I(λ) of labeled DNA upon excitation at wavelength $\lambda_{exc.}$= 480 nm. B. Fluorescence maximum at 530 nm I_{530} as derived from the spectra shown in A, plotted versus the DNA concentrations C_{DNA}.

The data shown in **Figure S15B** were fitted by linear regression analysis to:

$$I_{530}(C_{DNA}) = 282\ mL/ng \cdot C_{DNA} + 5794, \text{ leading to}$$

$$C_{DNA}(I_{530}) = (I_{530} - 5794)/(282\ mL/ng)$$

The calibration curve from **Figure S15B** was the one used to determine the DNA concentrations of the dissolved capsules. Dilutions (dilution factor f_{DF} = 1200) of dissolved capsules were mixed with the diluted RNA reagent (1:1 in vol.) and incubated for 5 min at room temperature. Then, the fluorescence spectra of the dissolved capsules mixed with the RNA reagent (1:1 in vol.) were measured and the concentration of encapsulated DNA was calculated, taking into account that capsules were diluted 1200 times. To illustrate this process, the following calculation is shown for the example of $(DEXS/PARG)_4$ capsules. Here, the maximum fluorescence peak at 530 nm for $(DEXS/PARG)_4$ capsules was measured to be I_{530} = 41530 a.u. Thus,

$$C_{DNA}(I_{530} = 41530) = (I_{530} - 5794) \cdot f_{DL}/(282\ mL/ng) =$$

$$(41530 - 5794) \cdot 1200 /(282\ mL/ng) = 148\ ng/\mu L.$$

As previously mentioned, the amount of biodegradable capsules per mL was roughly estimated with a hemocytometer, and reasonably assumed equal for all types of capsules ($C_{capsules} = 2 \cdot 10^8$ [caps/mL]). After similar calculations for all types of biodegradable capsules, the following concentrations for encapsulated DNA in the capsule stock solutions were obtained (**Table S2**).

Table S2. DNA concentration C_{DNA} of in the stock solutions of the different biodegradable capsules, and estimation of the amount of the loaded cargo per capsule.

Capsule architecture	C_{DNA} [ng/µL]	$C_{DEX\text{-}blue}$ [ng/µL]	$C_{capsules}$ [caps/mL]	m_{DNA} [g/caps]	$m_{DEX\text{-}blue}$ [g/caps]
SiO_2 (low TEOS)	110	39.6	$2 \cdot 10^8$	$55.0 \cdot 10^{-14}$	$19.8 \cdot 10^{-14}$
SiO_2 (high TEOS)	153	45.1	$2 \cdot 10^8$	$76.5 \cdot 10^{-14}$	$22.5 \cdot 10^{-14}$
$(DEXS/PARG)_4$	148	37.5	$2 \cdot 10^8$	$74.0 \cdot 10^{-14}$	$18.7 \cdot 10^{-14}$

Biodegradable capsules contain 3.35 ± 0.61 times (average ± standard deviation) more DNA than DEX-blue (in absolute weight). This means that a m_{DNA} = 50 pg/capsule approximately corresponds to $m_{DEX\text{-}blue}$ = 12-18 pg/capsule, and an average of 15 pg/capsule, for all the biodegradable capsules.

For the in vitro transfection, the capsules were added to ensure a DNA amount $m_{DNA/cell}$ of 50 pg of DNA per cell. Since we worked with a number of N = 40,000 cells/well (for transfection experiments), the total mass of DNA needed per well then was:

$$m_{DNA} = m_{DNA/cell} \cdot N_{cell} = 50 \text{ pg} \cdot 40000 = 2 \cdot 10^6 \text{ pg}.$$

The required volume of the capsule solution, illustrated for the $(DEXS\text{-}PARG)_4$ capsules, can then be calculated as:

$$V = m_{DNA}/C_{DNA} = 2 \cdot 10^6 \cdot 10^{-12} \text{g} / (148 \cdot 10^{-9} \text{g}/\mu\text{L}) = 13.5 \ \mu\text{L}.$$

Such figures would also correspond to around 15 pg DEX-blue per cell. Given that DNA is expected not to show appreciable toxic effects, with compared with DEX-blue, such estimation can be used to compare toxicity values.

8. Cell culture

Fetal bovine serum (FBS, Biochrom, #S0415), penicillin and streptomycin (P/S, Gibco, #15140-122), phosphate buffered saline (PBS, Biochrom, #L1825), Dulbecco's Modified Eagle's Medium (DMEM, Sigma,#D1145), trypsin/EDTA 0.05% solution (Gibco, Germany,#25300-054), 24-well plates (Corning, #CLS3527), 96-well plates (Corning, #CLS3526), FACS tubes (Falcon, Germany, #352052), and 8-well plates (Ibidi, #80826) were used for the cell culture.

Human cervical carcinoma cells (HeLa) were obtained from the American Type Culture Collection (ATCC) and were grown in Dulbecco's Modified Eagle's Medium supplemented with 10% FBS and 1% P/S at 37 °C in 5% CO_2.

9. Cytotoxicity studies

Cell viability studies were performed with a fluorescence-based approach using resazurin similar to previously published protocols.[9] Resazurin is a non-toxic non-fluorescent compound, which in living cells is converted into fluorescent resorufin.[10] 7,500 HeLa cells were seeded per well into 96-well plates. Each well contained 100 μL of medium per well and 0.32 cm^2 of surface area. After 24 h, the growth medium was replaced by medium (with and without FBS) containing SiO_2 (low TEOS), SiO_2 (high TEOS), $(DEXS/PARG)_4$, and $(PSS/PAH)_4$ loaded with DEX-blue capsules at different capsule concentrations. Cells were then incubated with capsules in the two following ways: (i) first 4 h of incubation in the cell growth medium without FBS, and then 20 h of incubation in the medium supplemented with FBS; (ii) 24 h of incubation in the cell growth medium supplemented with FBS. After incubation, cells were washed with PBS and incubated with 10% vol. of fresh resazurin solution in pure growth media was added. After 4 hours, the samples were analyzed with a fluorimeter ($\lambda_{exc}/\lambda_{em}$ = 560 nm/575 nm). For the evaluation the background-corrected maximum of the fluorescence emission was used, since this emission correlates

with the viability V of cells [9,11]. Each measurement was repeated three times to obtain the mean value and the standard deviation. The mean value of the fluorescence intensity was normalized to the fluorescence of cells that had not been exposed to capsules, and was plotted against the concentration of the capsules (**Figures S16 - S19**).

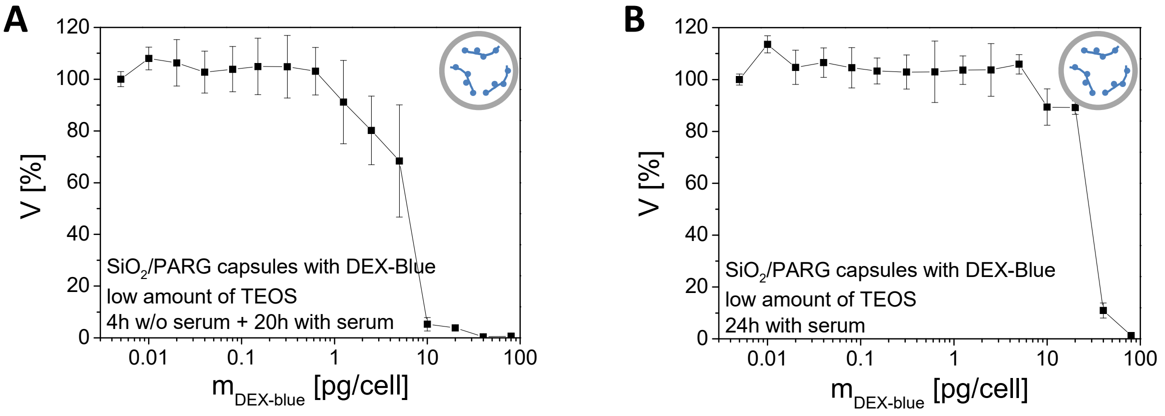

Figure S16. Viability V of HeLa cells upon exposure to SiO_2 (low TEOS) capsules loaded with DEX-blue at the amount of $m_{DEX\text{-}blue}$. A. After incubation for 4 h in FBS free medium and for 20 h in complete medium. B. After incubation for 24 h in complete medium.

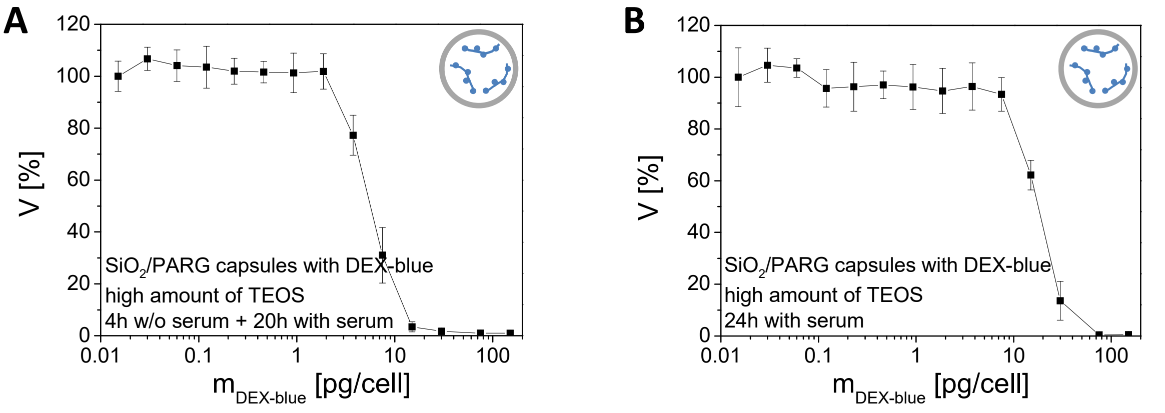

Figure S17. Viability V of HeLa cells upon exposure to SiO_2 (high TEOS) capsules loaded with DEX-blue at the amount of $m_{DEX\text{-}blue}$. A. After incubation for 4 h in FBS free medium and for 20 h in complete medium. B. After incubation for 24 h in complete medium.

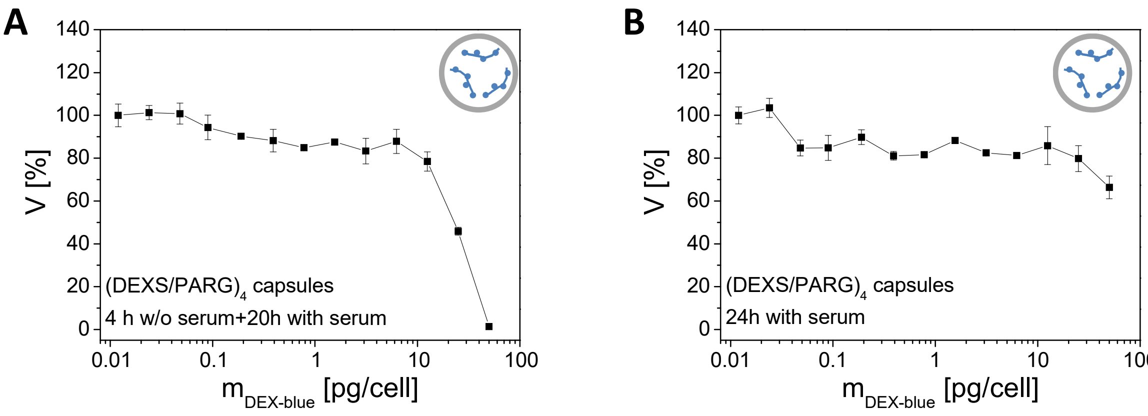

Figure S18. Viability V of HeLa cells upon exposure to $(DEXS/PARG)_4$ capsules loaded with DEX-blue at the amount of $m_{DEX\text{-}blue}$. A. After incubation for 4 h in FBS free medium and for 20 h in complete medium. B. After incubation for 24 h in complete medium.

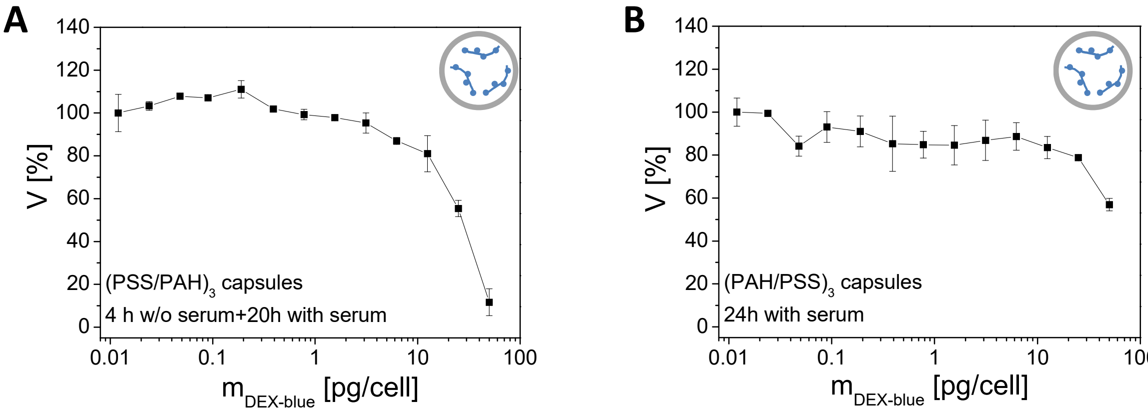

Figure S19. Viability V of HeLa cells upon exposure to $(PSS/PAH)_4$ capsules loaded with DEX-blue at the amount of $m_{DEX\text{-}blue}$. A. After incubation for 4 h in FBS free medium and for 20 h in complete medium. B. After incubation for 24 h in complete medium.

Additionally, the cell viability of SiO_2 capsules (low and high TEOS) co-loaded with DNA and DEX-blue was examined (**Figures S20-S21**).

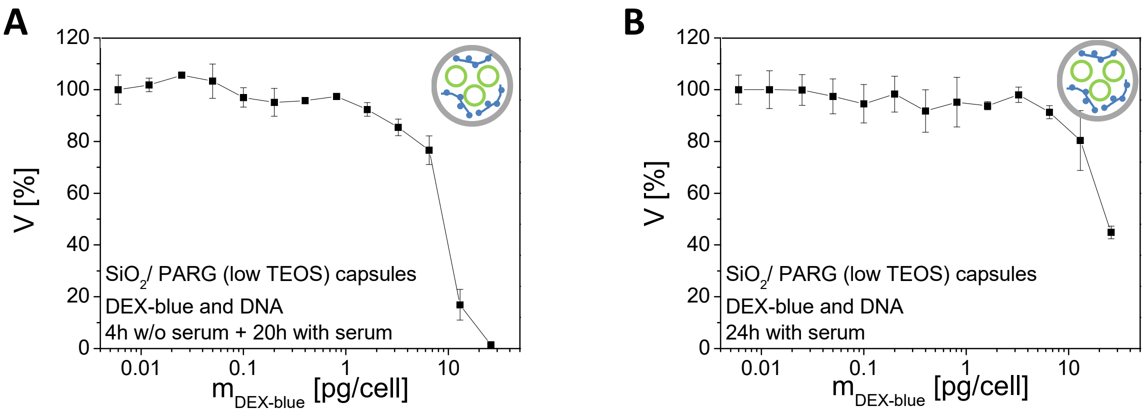

Figure S20. Viability V of HeLa cells upon exposure to SiO_2 (low TEOS) capsules co-loaded with DNA and DEX-blue at the amount of m_{DNA} and $m_{DEX\text{-}blue}$. It should be considered that an amount of m_{DNA} = 50 pg/cell approximately corresponds to $m_{DEX\text{-}blue}$ = 12-18 pg/cell, average 15 pg/cell. A. After incubation for 4 h in FBS free medium and for 20 h in complete medium. B. After incubation for 24 h in complete medium.

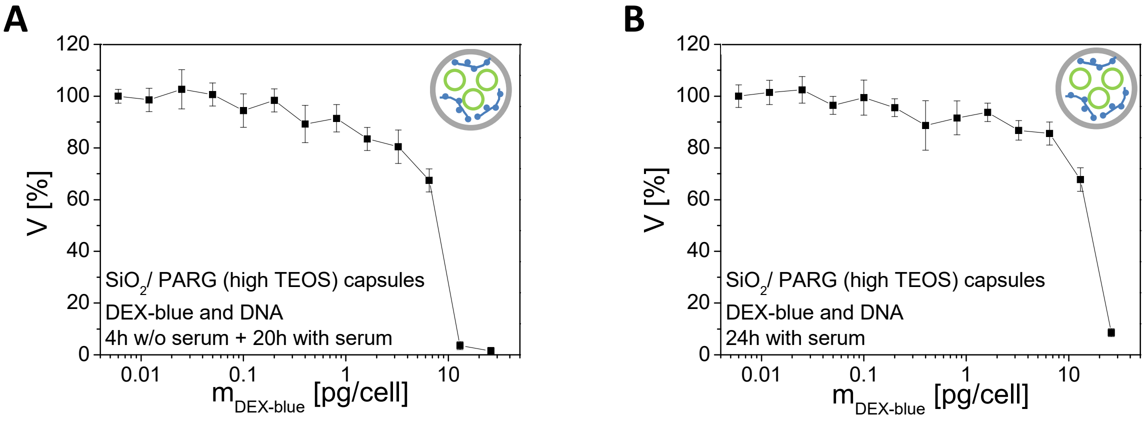

Figure S21. Viability V of HeLa cells upon exposure to SiO_2 (high TEOS) capsules co-loaded with DNA and DEX-blue at the amount of m_{DNA} and $m_{DEX\text{-}blue}$. It should be considered that an amount of m_{DNA}=50 pg/cell approximately corresponds to $m_{DEX\text{-}blue}$=12-18 pg/cell, average 15 pg/cell. A. After incubation for 4 h in FBS free medium and for 20 h in complete medium. B. After incubation for 24 h in complete medium.

10. Uptake studies

To quantify the uptake of capsules by cells, SiO_2 (low and high TEOS), $(DEXS/PARG)_4$, and $(PSS/PAH)_4$ capsules loaded with Dex-blue were added to HeLa cells. Cells were seeded at a density of 40,000 cells/well in 24-well plates (500 µL of medium added per well, 1.9 cm^2 surface area per well). On the next day, capsules of each type were added to the cells at the amount of $m_{DEX\text{-}blue}$ = 1.5 and 3 pg (DEX-blue) per cell. In **Figure 3A** microscopy images of cells with associated capsules are shown. Note that some

capsules may stick only to the outer cell membrane, while most others will have been internalized by cells [12].

HeLa cells were incubated with the different types of capsules at 37 °C, 5% CO_2 for different time intervals. Moreover, the conditions of incubation were varied. First, cells were incubated with the capsules (i) in the cell culture medium supplemented with FBS for 2, 4, 6, and 24 h; (ii) in the cell culture medium without FBS for 2, 4, and 6 h; (iii) first 2 h in the cell culture medium without FBS and then the medium was replaced with the cell culture medium supplemented with FBS; (iv) first 4 h in the cell culture medium without FBS and then the medium was replaced with the cell culture medium supplemented with FBS. After incubation, cells were washed 3 times with PBS and detached with 100 µL of trypsin-EDTA. Trypsin was then neutralized with 400 µL of cell culture medium supplemented with FBS. Detached cells with associated capsules were collected into the FACS tubes and centrifuged for 5 minutes at 220xg. The supernatant with non-internalized capsules was discarded and the cells with associated capsules were dispersed in 500 µL of PBS. Note, that this washing procedure should have removed also a significant amount of capsules sticking only to the outer cell membrane, but this removal will not be quantitative. Still, despite being aware that still some capsules associated with cells may actually not be internalized, in the following we will refer to internalized capsules. A more sophisticated approach could have involved loading of the capsules with pH-sensitive fluorophores [12-14]. Cells with internalized capsules were then analyzed by flow cytometry (LSRFortessa, equipped with a diode laser (405 nm)). Forward and side scattering signals (FSC, SSC, in which both, the area (A) and the width (W) of the scattering peak were analyzed) were used to exclude debris and cell duplets by appropriate gating, leaving only the population with single cells subject to analysis (**Figure S22**).

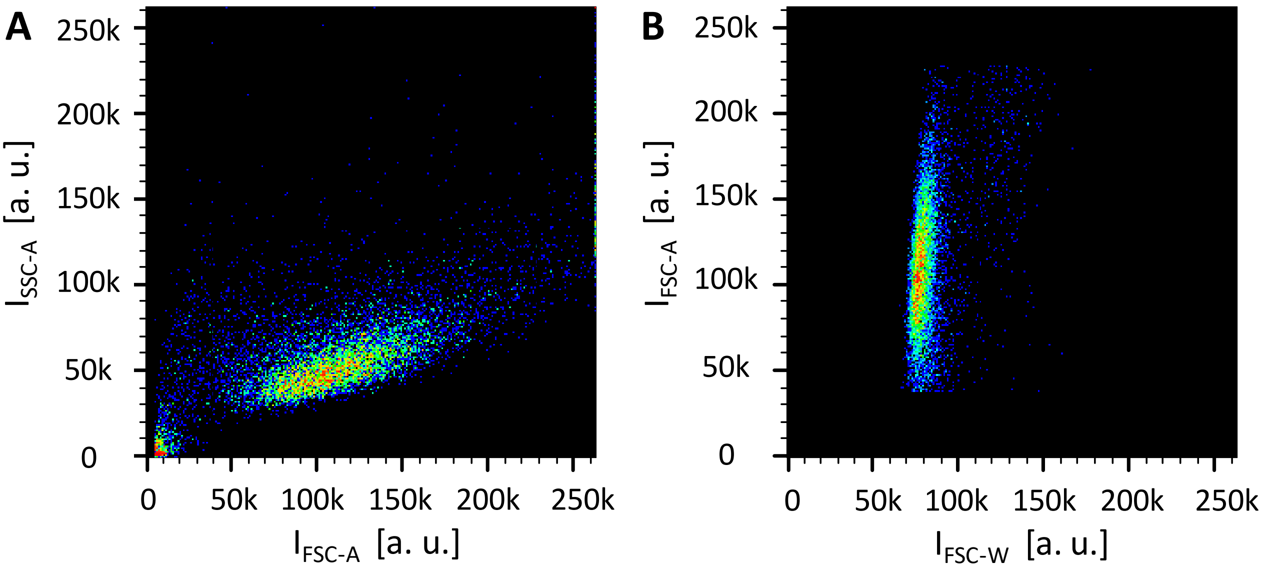

Figure S22. Two dimensional density plots of untreated HeLa cells. A. Side-area scattering intensity, I_{SSC-A} versus forward-are scattering intensity, I_{FSC-A}. The events with low side-area and forward-area scattering correspond to the cell debris and can be excluded by gating G1. B. Forward-area scattering intensity, I_{FSC-A}

versus forward-width scattering intensity I_{FSC-W}. The events with high forward-scattering width correspond to cell duplets and can be excluded by gating G2. The number of events is color-coded. The same gatings were used for all cells incubated with capsules.

Besides the side- and forward-scattering signals, the blue fluorescence originating from internalized capsules was also recorded for all events. This fluorescence I_{blue} corresponded to cells associated with capsules. The blue fluorescence intensity distribution $N(I_{blue})$ is plotted in **Figure S23**.

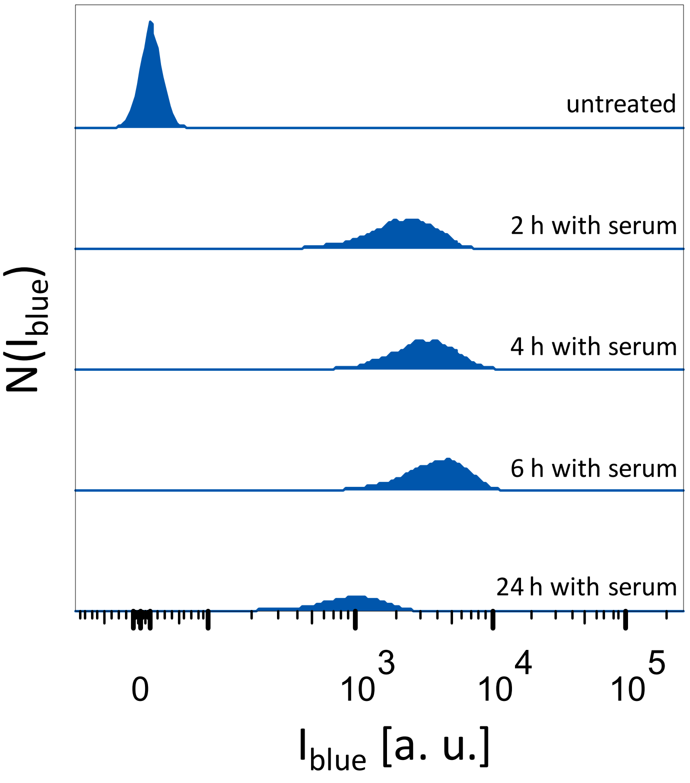

Figure S23. Example of the distribution of blue fluorescence intensity $N(I_{blue})$ of untreated cells (without incubation with capsules), and of HeLa cells, which had been incubated with SiO_2 (low TEOS) for 2, 4, 6, and 24 h.

From the blue fluorescence distributions (**Figure S23**), the mean fluorescence intensity $\langle I_{blue} \rangle$ of the blue signal coming from each cell was calculated and plotted versus the conditions of incubation for SiO_2 (low and high TEOS), $(DEXS/PARG)_4$, and $(PSS/PAH)_4$ capsules (**Figures S24 - S25**). The auto-fluorescence from untreated cells was subtracted by counting the fluorescence exceeding the auto-fluorescence signal.

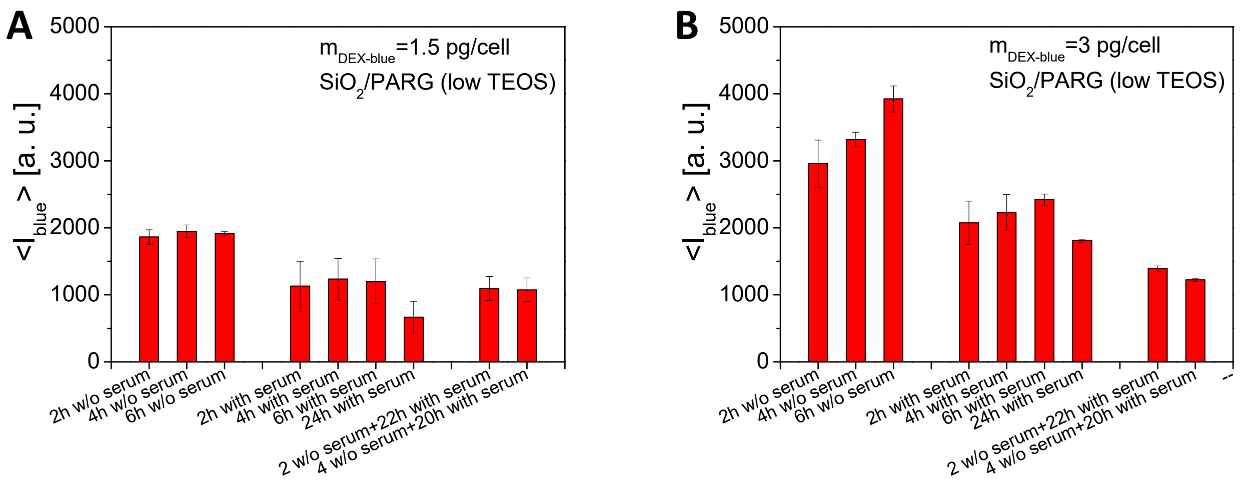

Figure S24. Mean blue fluorescence intensity <I$_{blue}$> of HeLa cells after incubation with DEX-blue-loaded SiO_2 (low TEOS) capsules at different conditions of incubation. About 10,000 events were analyzed. The error bars correspond to the standard deviation from 3 independent experiments.

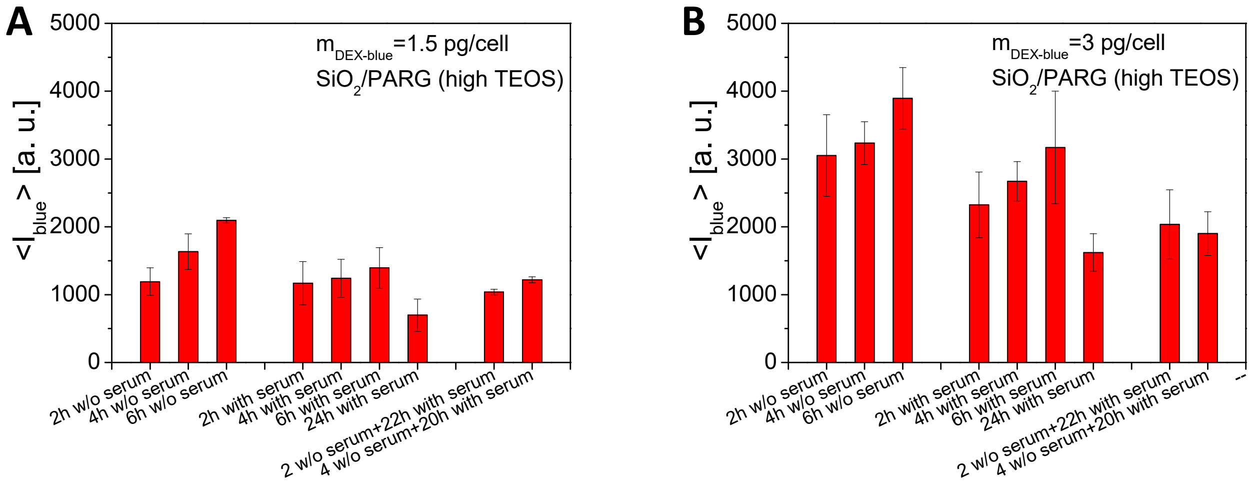

Figure S25. Mean blue fluorescence intensity <I$_{blue}$> of HeLa cells after incubation with DEX-blue-loaded SiO_2 (high TEOS) capsules at different conditions of incubation. About 10,000 events were analyzed. The error bars correspond to the standard deviation from 3 independent experiments.

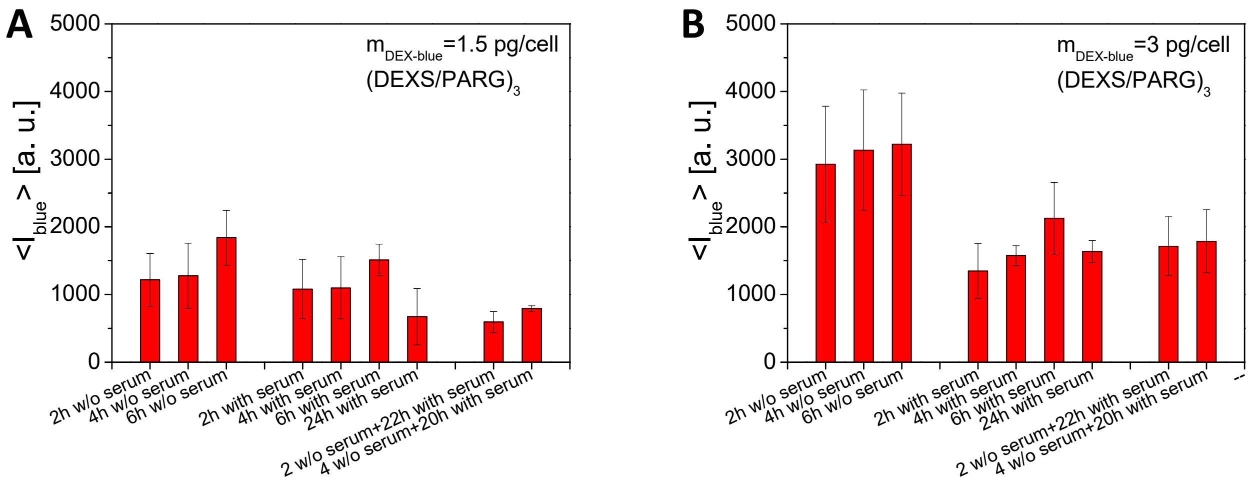

Figure S26. Mean blue fluorescence intensity <I_{blue}> of HeLa cells after incubation with DEX-blue-loaded (DEXS/PARG)$_4$ capsules at different conditions of incubation. About 10,000 events were analyzed. The error bars correspond to the standard deviation from 3 independent experiments.

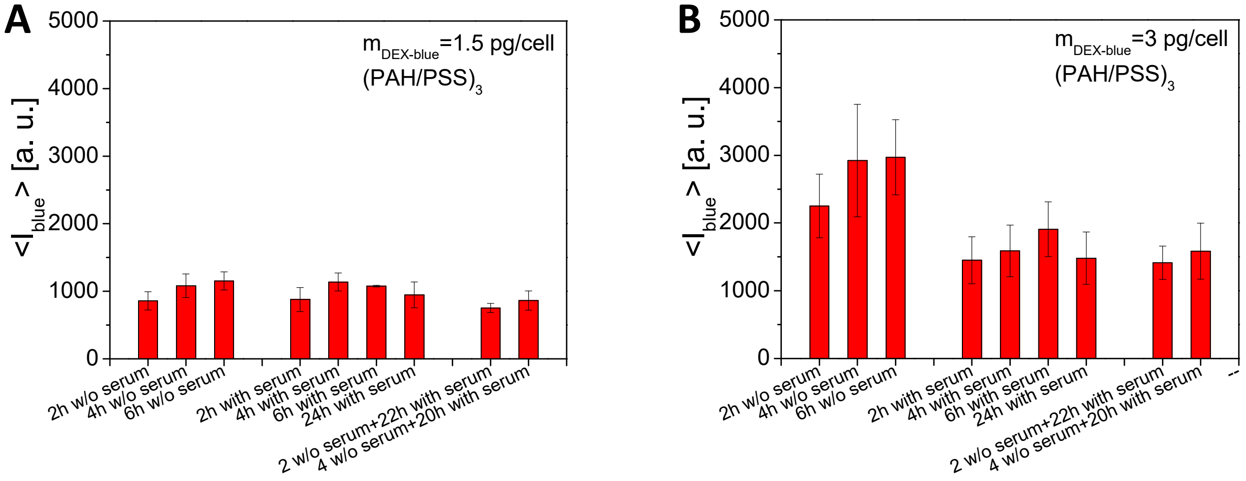

Figure S27. Mean blue fluorescence intensity <I_{blue}> of HeLa cells after incubation with DEX-blue-loaded (PSS/PAH)$_4$ capsules at different conditions of incubation. About 10,000 events were analyzed. The error bars correspond to the standard deviation from 3 independent experiments.

11. Proliferation studies

The effect of SiO_2 capsules (low and high TEOS) on the cell proliferation was studied by directly measuring the DNA synthesis according to previously published protocols.[9] This can be quantified by the incorporation of the thymidine-analog EdU (5-ethynyl-2′-deoxyuridine). The detection of EdU can be made by a copper-catalyzed click-reaction between its alkyne group and an azide group-containing fluorophore.

HeLa cells were seeded into 96-well plates (5,000 cells/well) and left overnight. Afterwards, the growth medium was exchanged with the medium containing SiO_2 (low and high TEOS) capsules loaded with DEX-AF647 at different concentrations ($m_{DEX-AF647}$ = 0-3 pg/cell). Colchicine (Sigma Aldrich, #C3915), a compound suppressing cell proliferation via inhibition of chromosome segregation during mitosis[15] was used at a concentration of 10 μM as negative control. HeLa cells were incubated with the different types of capsules for (i) 4 h in the medium without FBS followed by 20 h in medium supplemented with FBS, (ii) 24 h in medium supplemented with FBS. After incubation, the growth cell medium was replaced by medium containing 10 μM EdU (Molecular Probes, #A10044) for additional 6 h. Cells were then fixed with 50 μL of 100% methanol for 15 min at 4 °C, and permeabilized with 50 μL of a solution of 0.5 mg/mL saponin and 5 mg/mL glycine in PBS for 5 min. In order to label the incorporated EdU with Alexa Fluor

488 azide (Molecular Probes, #A10266), 1.7 M Tris buffer pH 8.5 (Sigma Aldrich, #T1503), 100 mM $CuSO_4$ (Sigma Aldrich, #61230), 10 mM Alexa Fluor 488 azide, and 500 mM ascorbic acid (Sigma Aldrich, #255564) were added to the cells for 30 min at 37 °C.[9] Additionally, the nuclei of cells were stained with 5 μM DAPI (Molecular Probes, #D1306) for 10 min.

Imaging was performed with a wide field microscope (Zeiss, Axiovert 200M). Imaging conditions were λ_{exc} = 480±15 nm, beam splitter at 505 nm, and λ_{em} = 535±20 nm for the green channel, in order to visualize EdU-Alexa Fluor 488. The filter cube Zeiss DAPI Set 49 was used for detecting DAPI (λ_{ex} = 365 nm, beam splitter at 395 nm, and λ_{em} = 445±25 nm, for the blue channel. The imaging area for each well was around 10 mm^2. Fluorescence micrographs were used to estimate the proliferation rate p.[9] These micrographs were processed using MATLAB (Mathworks) software and Cell profiler in the following way:[9] DAPI-stained nuclei were segmented, representing proliferated and non-proliferated cells present in the current micrograph. Afterwards, the mean fluorescence intensity in the blue channel (I_{DAPI}) per nucleus was determined, as well as the mean intensity of EdU-Alexa Flour 488 in the corresponding green channel (I_{EdU}). For each nucleus the logarithm of both intensity values was plotted in a scatter plot to allowed for identification and gating of proliferated cells from non-proliferated ones (**Figure S28**) [9]. Finally, the fraction p of proliferated cells from all cells was calculated for each image and averaged for each concentration. **Figure S29** shows the mean values ± standard deviations resulting from three individual experiments [9].

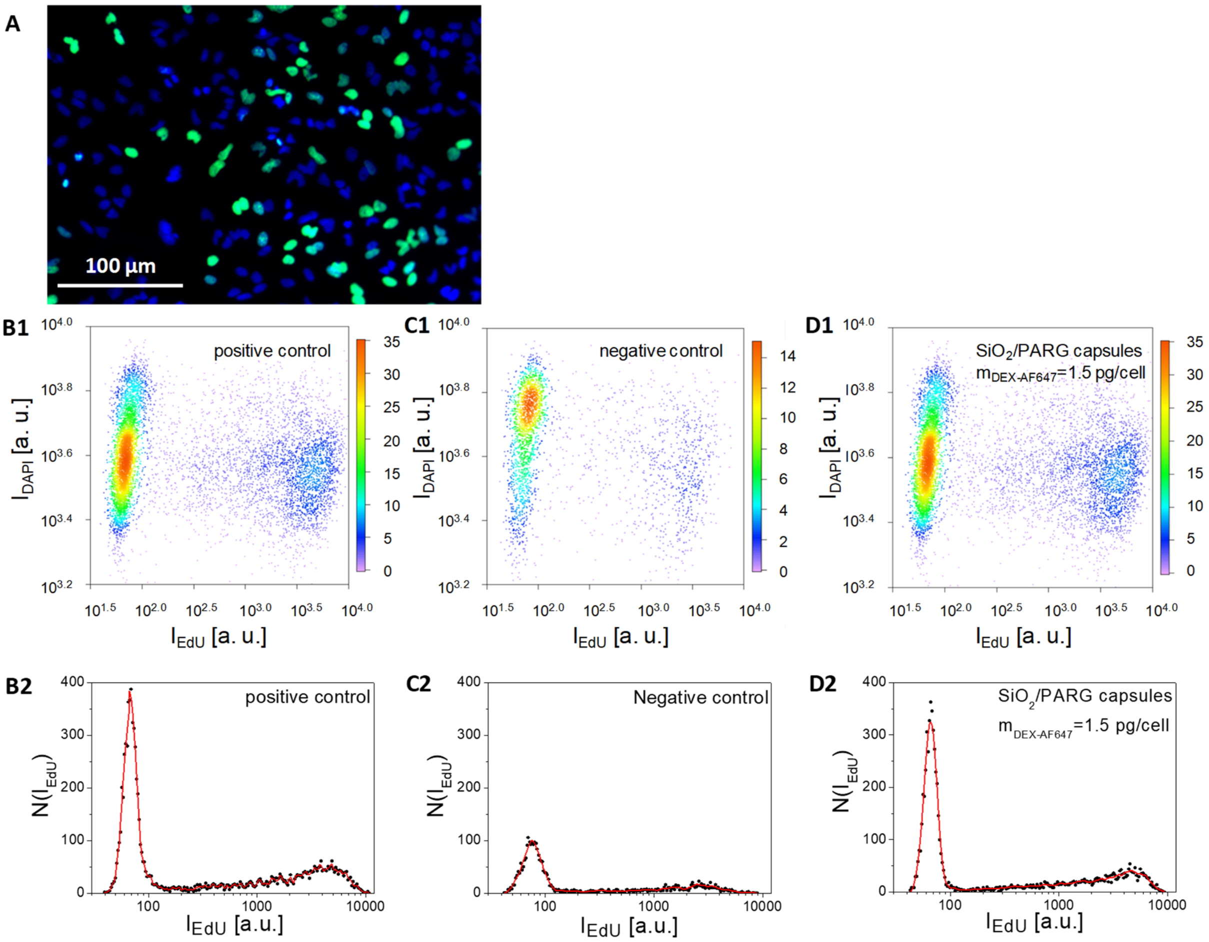

Figure S28. Cell proliferation assay carried out with SiO_2/PARG (low and high TEOS) capsules loaded with DEX-AF647. A. Example of a fluorescence image used to derive the quantitative results. This fluorescence image is shown for HeLa cells incubated for 24 h with SiO_2/PARG (low TEOS) capsules at the amount of $m_{DEX-AF647}$ = 1.5 pg/cell. The nuclei of the cells are stained with DAPI (blue), proliferated cells show additional green signal. The color-coded 2D density plots show 2 populations: the left population corresponds to the non-proliferating cells; the right population corresponds to the proliferating cells. Data are shown for B1. positive control, C1. negative control, D1. SiO_2 (low TEOS) capsules at $m_{DEX-AF647}$ = 1.5 pg/cell. B2 - D2 show the corresponding distribution functions.

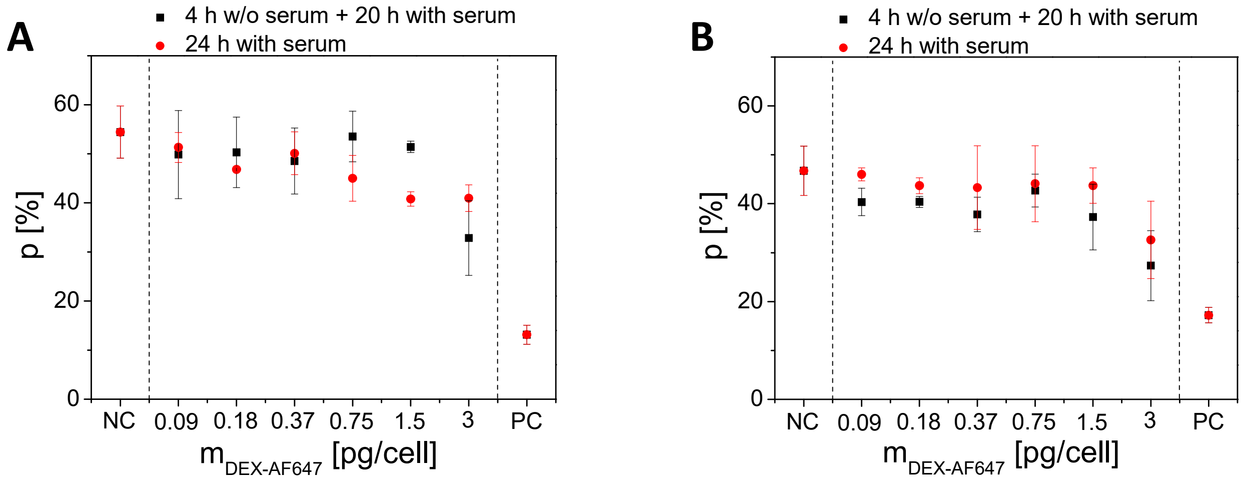

Figure S29. Proliferation rate p of cells after exposure to (A) SiO_2 (low TEOS), (B) SiO_2 (high TEOS) capsules.

12. Capsule degradation

In order to trigger the mechanism of the protein expression upon delivery of DNA plasmids, the DNA has to be delivered to the nuclei of cells. It is known that capsules are internalized by cells and afterwards they are located inside the endocytic vesicles [16] and, thus, not free in the cytosol. These vesicles have different environment than the cytosol and their membrane is permeable neither for many types of delivered cargo nor the products of the capsule degradation [17]. Thus, it is important to overcome this barrier and to deliver the genes into the cytosol of cells, that they can reach the nucleus.

In order to observe the degradation of SiO_2 capsules they were loaded with the commercially available protein ovalbumin, which was saturated with BODIPY dye [8]. In its non-degraded state, DQ-OVA is self-quenched and yields fluorescence signal in the red region of spectrum. This fluorescence is coming from the dye dimmers (640 nm).[7] As soon as DQ-OVA starts to degrade, the distance between the dye molecules increases and the protein thus gives bright green fluorescence (510 nm) [18].

Confocal microscopy (Zeiss, CLSM Meta 510) was used to observe the capsules. Capsules loaded with DQ-OVA were dispersed in water and an argon laser (488 nm) was used to excite the capsules with a Plan-Apochromat 63x/1.40 Oil DIC M27 objective. The green fluorescence was collected using a band pass (BP) filter from 505-580 nm and the red fluorescence was recorded using a longpass (LP) filter of 615 nm. As it can be observed in **Figure S30**, non-degraded capsules have fluorescence signal in the red and green regions of the spectrum. The red signal comes from the quenched dye dimmers, whereas a weak green signal comes from non-quenched dye molecules. Both signals are colocalized.

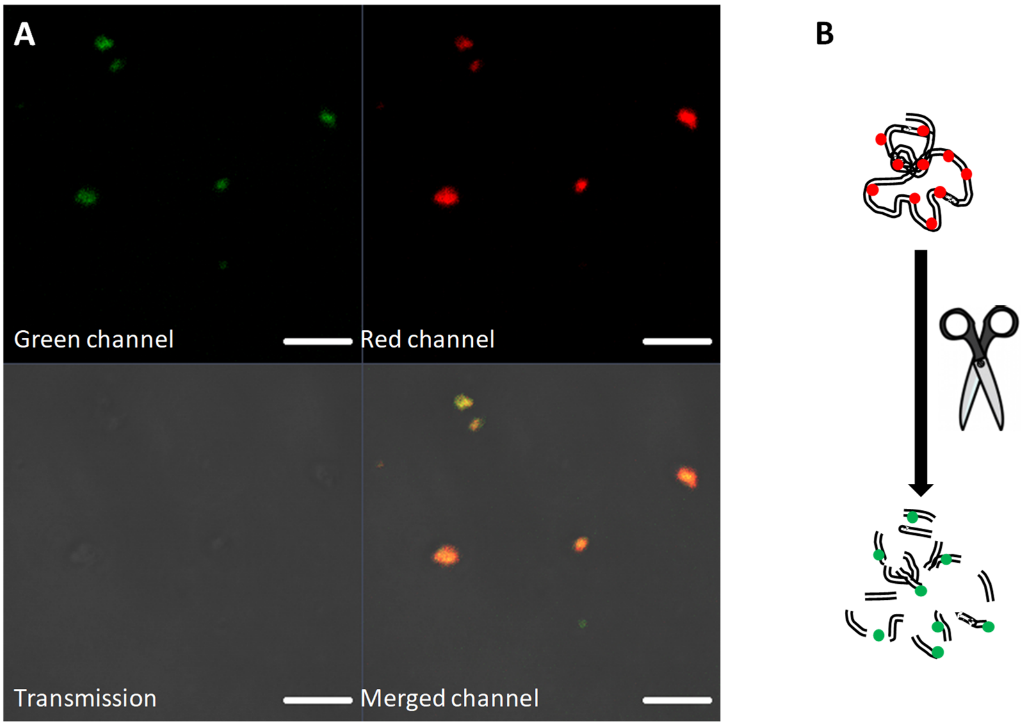

Figure S30. A. Representative confocal microscopy images of SiO_2 (low TEOS) capsules loaded with DQ-OVA. In the green and red channels, an argon laser at 488 nm was used as excitation source. For the green channel a band pass filter (505-580 nm), for the red channel a long pass filter (615 nm) was used. The scale bar corresponds to 20 µm. B. Scheme for the enzymatic cleavage of DQ-OVA, as discussed previously [8]. The sketch is adopted from Rivera Gil et al [8].

In order to observe the degradation of SiO_2 capsules, HeLa cells were seeded into 8-well plates (ibidi) at an amount of 25,000 cells/well, with 250 µL of medium added per well, 1 cm^2 surface area per well. The next day, SiO_2 capsules as prepared with low amount of TEOS were added to the cells in each well (2 µL of the capsule stock solution) and HeLa cells were incubated with capsules for 24 hours. Afterwards, cells were observed under CLSM (**Figure 4B**).

13. Stability of DNA plasmids

Transfection efficiency of cells can be influenced by different factors. One of them is the degradation of the genetic material, here plasmids encoding GFP (green fluorescent protein, pEGFP-N1, 4700 base pairs) in the intracellular environment. In order to mimic lysosomes, pronase at concentration of 2 mg/mL was used. To study the degradation, plasmids were transferred into (i) PBS at pH 4, (ii) pronase at pH 7, (iii) pronase pH 4, (iv) PBS at pH 7 and placed at 37 °C, 5% CO_2 overnight. Afterwards, the commercially available transfection reagent Lipofectamine 2000 (Invitrogen, #11668-030) was used to transfect cells with the treated plasmids. The transfection of cells with Lipofectamine 2000 was performed according to the protocol (https://tools.thermofisher.com/content/sfs/manuals/Lipofectamine_2000_Reag_protocol.pdf).

In order to reach optimal transfection conditions, high and low amounts of Lipofectamine Reagent was used. The differently treated DNA plasmids were mixed with the Lipofectamine reagent (low and high amount). The ratio Lipofectamine:DNA (C_{DNA} =1.8 mg/mL)=2:1 μL/μL was used for the low amount, whereas the ratio Lipofectamine:DNA (C_{DNA}=1.8 mg/mL)=4:1 μL/μL was used for the high amount. Afterwards, DNA-Lipofectamine mixtures were added to the cells at the amount m_{DNA} = 6.25, 12.5, 25, 50 pg/cell. Cells were placed in the incubator for at least 24 hours. After incubation, cells were prepared for flow cytometry analysis similar as in the uptake study (see §10 of this **Supporting Information**). Besides forward and side scattering signals, green fluorescence due to expressed GFP was recorded using an LSRFortessa flow cytometer equipped with a HeNe laser (488 nm). Gatings were used for the side and forward scattering signals (**Figure S31**) in order to get rid of signal from cell debris and cell duplets. Green fluorescence signal only from single cells was collected, and green fluorescence intensity distributions for all types of transfected cells were then plotted (**Figure S32**).

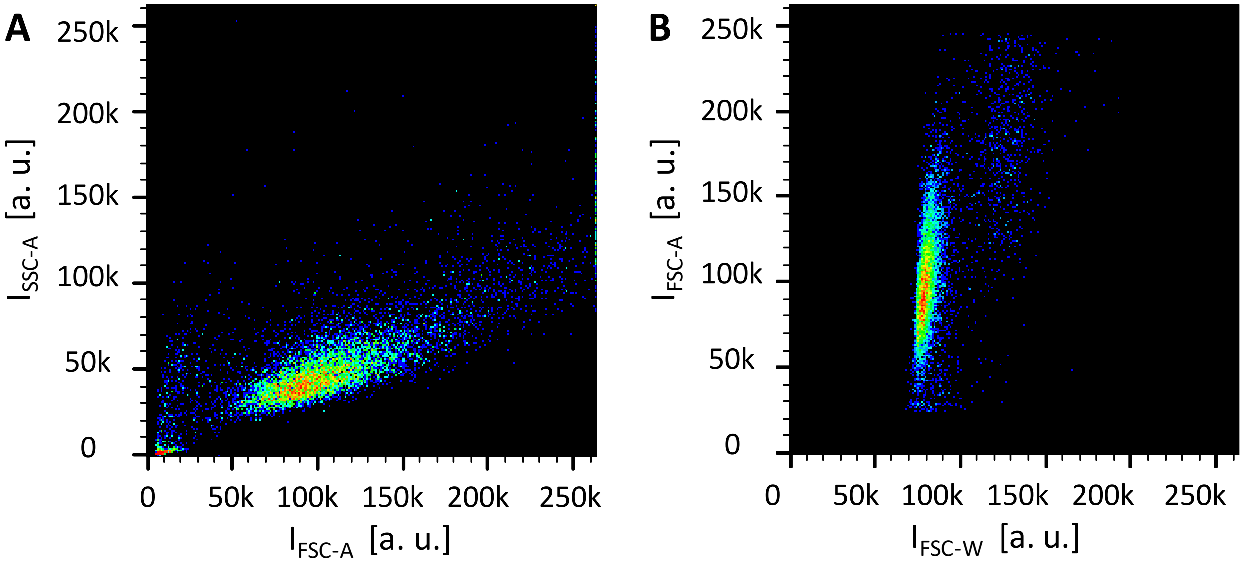

Figure S31. Two dimensional density plots of untreated HeLa cells. A. Side-area scattering intensity, I_{SSC-A} versus forward-area scattering intensity I_{FSC-A}. The events with low side-area and forward-area scattering correspond to cell debris are excluded by gating G1. B. Forward-area scattering intensity I_{FSC-A} versus forward-width scattering intensity I_{FSC-W}. The events with high forward-scattering width correspond to cell duplets and are excluded by gating G2. The number of events is color coded. The same gatings were used for all cells incubated with capsules.

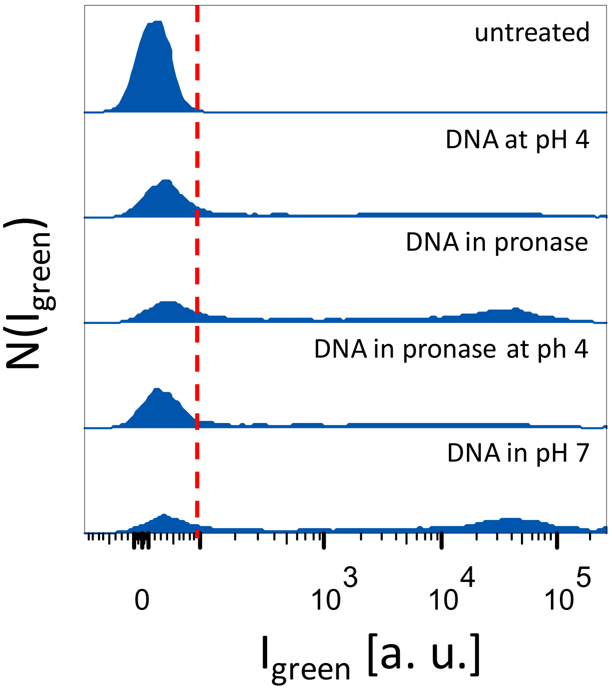

Figure S32. Example the distribution N(I_{green}) of green fluorescence intensity per cell I_{green} of untreated cells (without incubation with capsules), and of HeLa cells, which had been transfected with differently treated DNA.

From the plots shown in **Figure S32** the percentage of transfected cells was derived (**Figure S33**). Each event in which the green fluorescence signal was above the green signal intensity of untreated cells was considered as transfected cell. Experiments were repeated 3 times. The mean percentage of transfected cells T (and its standard deviation) was calculated and plotted.

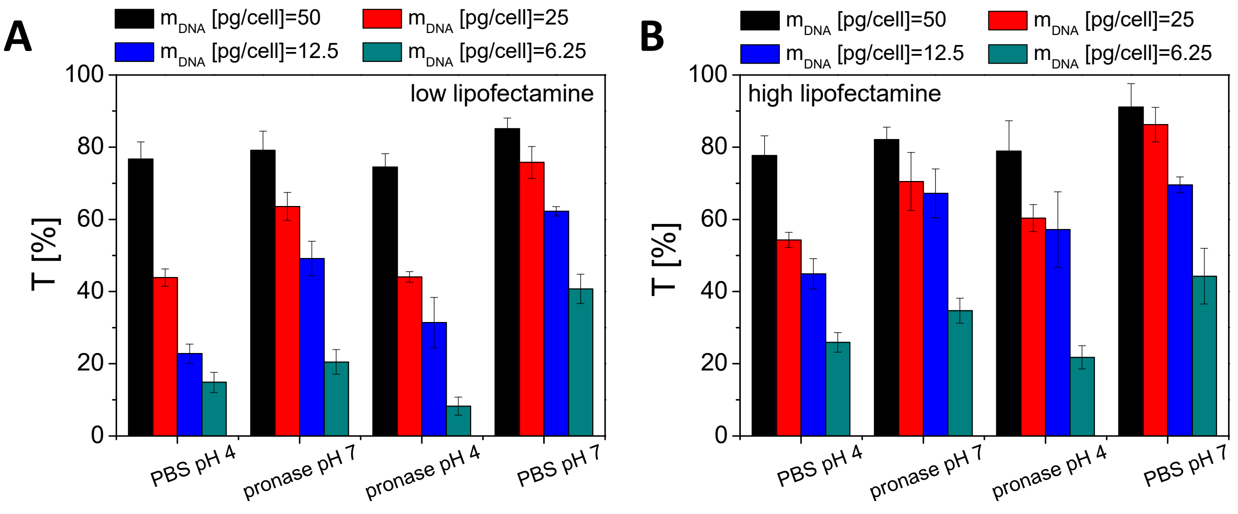

Figure S33. Fraction T of HeLa cells, which were transfected with differently treated DNA. The transfection reagent Lipofectamine 2000 was used to transfect cells at A. low amount and B. high amount of Lipofectamine 2000. DNA was added to the cells at the various amounts m_{DNA}. About 10,000 events were analyzed. The error bars correspond to the standard deviation from 3 independent experiments.

According to the obtained data, it can be concluded that the surrounding environment affects the ability of plasmid DNA that is used to transfect cells. In detail, untreated DNA showed the maximum of transfection efficiency and transfection was dose-dependent: more added DNA resulted in a higher rate of transfected cells. Acidic environment and the presence of pronase decrease the transfection efficiency of cells, probably due to the degradation of plasmids. Thus, since the pH value of endocytic vesicles is reported to be acidic [19,20], the possible degradation of the plasmid cargo can already start in these compartments.

14. In vitro transfection studies

HeLa cells were transfected with both SiO_2 (low and high TEOS), $(DEXS/PARG)_4$ and $(PSS/PAH)_4$ capsules co-loaded with plasmids (encoding form GFP) and DEX-blue (used as a reference). For this, HeLa cells were seeded into 24-well plates at an amount of 40,000 cells/well (surface area per well: 1.9 cm^2; medium per well: 0.5 mL). The next day, capsules were added to the cells at different amounts (m_{DNA} = 0 - 50 pg/cell). Non-degradable capsules were added to the cells at $m_{DEX\text{-}blue}$ = 1.5 - 12 pg/cell. Capsules were then incubated with cells (i) for 4 h in medium without FBS and then for 20 h in medium supplemented with FBS; (ii) for 24 h in medium supplemented with FBS; (iii) for 4 h in medium without FBS and then for 44 h in medium supplemented with FBS; (iv) for 48 h in medium supplemented with FBS. Additionally, in order to increase the pH values of lysosomes, which decreases the DNA degradation in acidic environment and lowers the activity of lysosomal enzymes, 7 μM chloroquine was added to the cells[21] (in cases when FBS free medium was replaced by medium with FBS after 4 h of incubation, additional 2 μM chloroquine was added).

After incubation, 24-well plates were placed under a confocal microscope (Zeiss, CLSM Meta 510). An argon laser at 488 nm was used as excitation source. For GFP detection (due to the delivered plasmid coding for GFP), an emission LP filter at 505 nm was used. Each picture was taken with a Plan-Apochromat 10x/0.45 objective (**Figures S34-S37**).

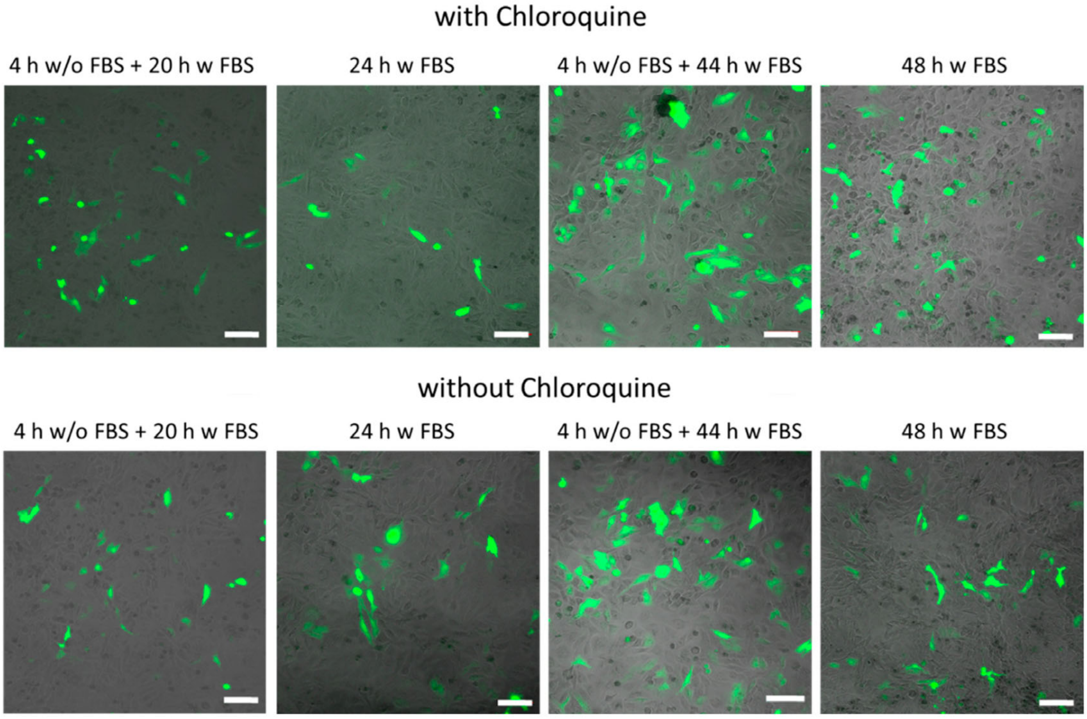

Figure S34. GFP expression observed by confocal microscopy of SiO_2 (low TEOS) capsules in HeLa cells transfected using different strategies. The added amount of capsules was m_{DNA} = 25 pg/cell. The scale bars correspond to 100 µm.

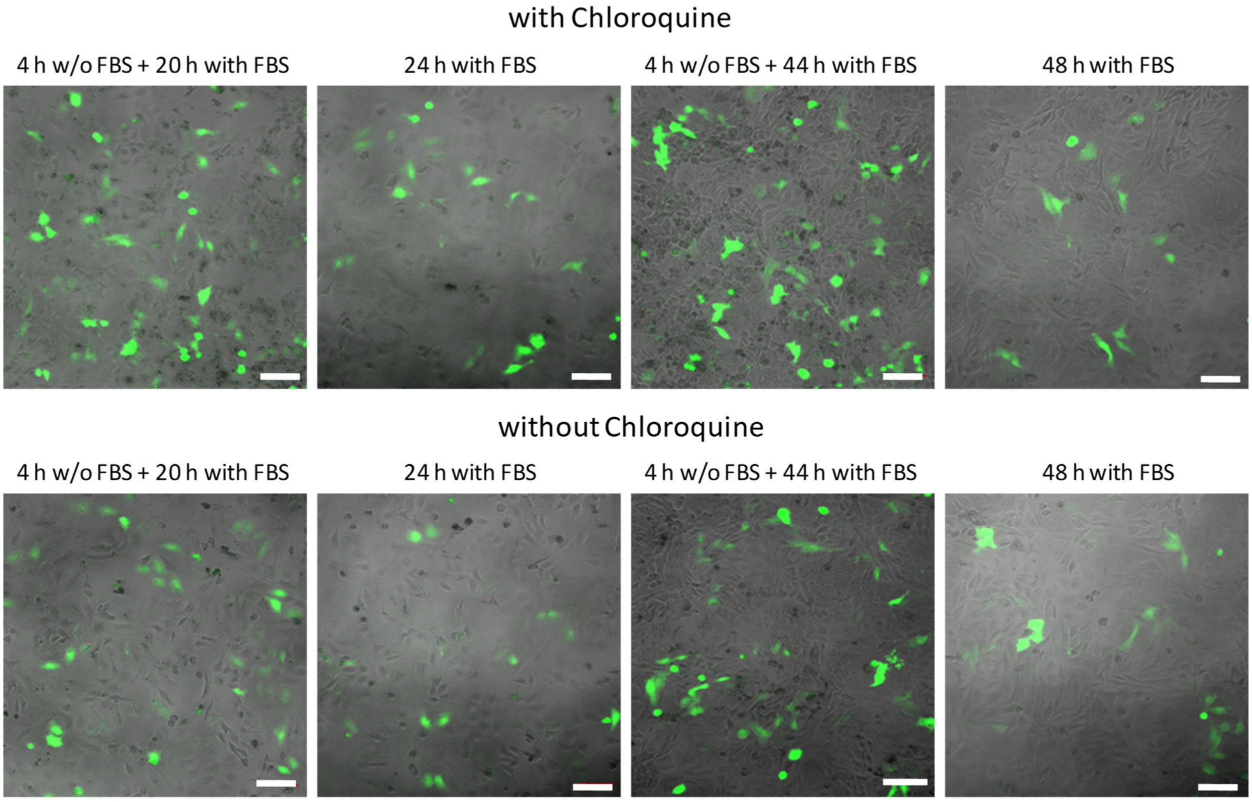

Figure S35. GFP expression observed by confocal microscopy of SiO_2 (high TEOS) capsules in HeLa cells transfected using different strategies. The added amount of capsules was m_{DNA} = 25 pg/cell. The scale bars correspond to 100 µm.

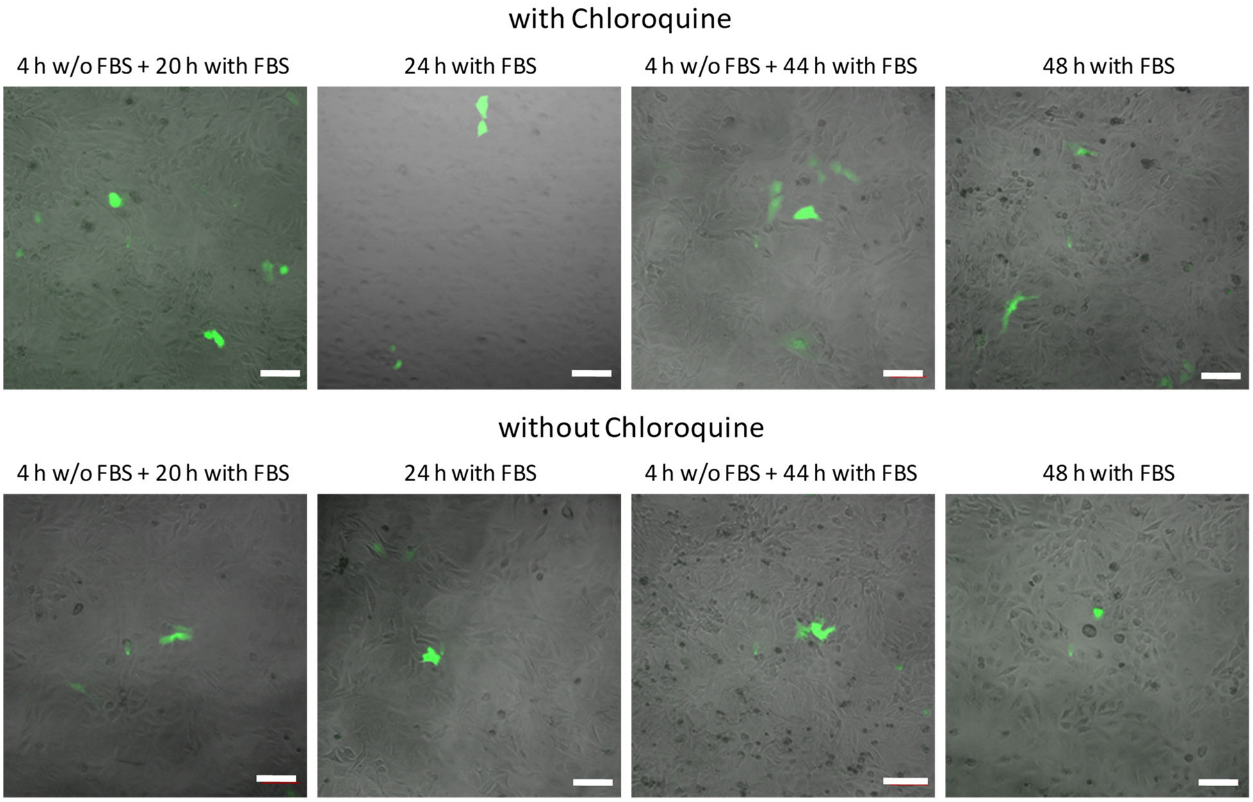

Figure S36. GFP expression observed by confocal microscopy of $(DEXS/PARG)_4$ capsules in HeLa cells transfected using different strategies. The added amount of capsules was m_{DNA} = 25 pg/cell. The scale bars correspond to 100 μm.

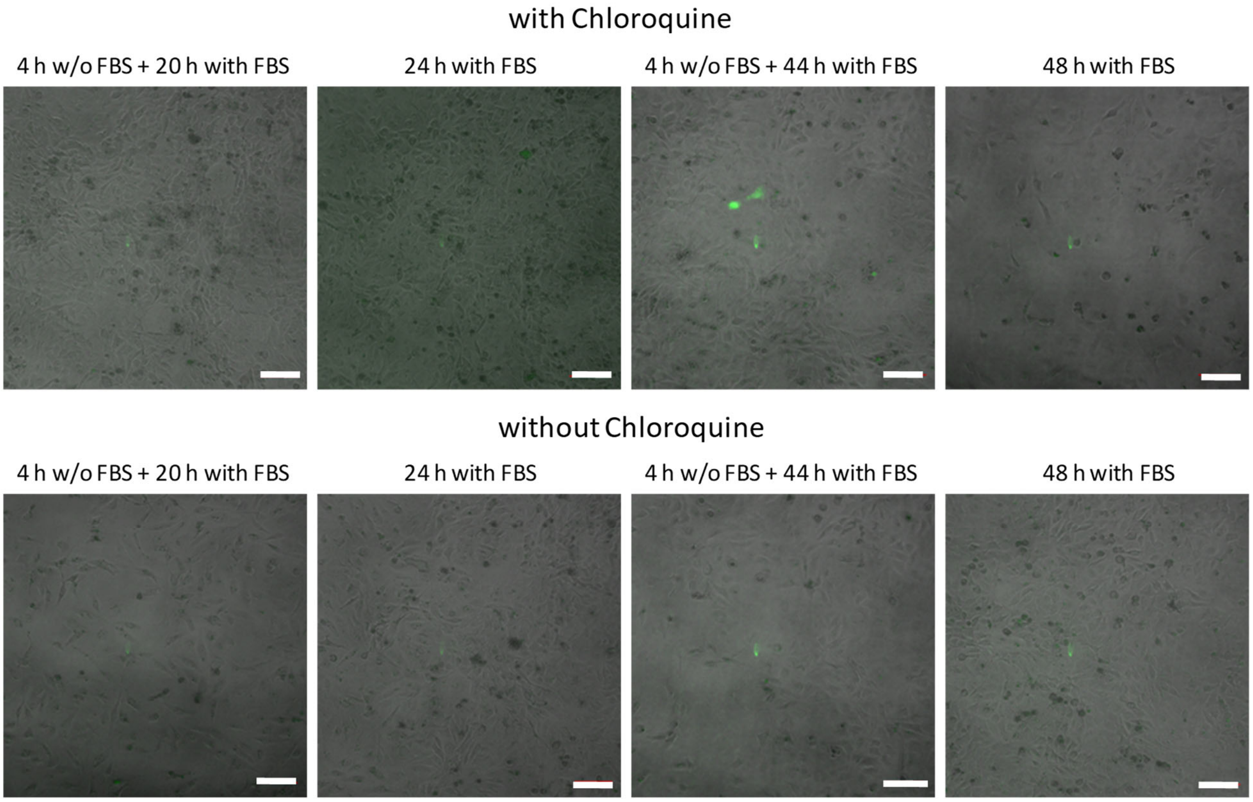

Figure S37. GFP expression observed by confocal microscopy of $(PSS/PAH)_4$ capsules in HeLa cells transfected using different strategies. The added amount of capsules was $m_{DEX\text{-}blue}$ = 6 pg/cell. The scale bars correspond to 100 μm.

Flow cytometry was also used for the quantitative analysis of the GFP expression. Cells were washed three times and trypsinized, as described in §10 of this **Supporting Information**. Afterwards, 400 μL of complete cell medium was added to each well and cells were transferred into FACS tubes. Cells were spun down at 220xg for 5 min. The supernatant was discarded, and the obtained pellet was dispersed in 400 μL of PBS. This solution was then subject to flow cytometry.

Uptake and transfection efficiency were analyzed with a LSRFortessa flow cytometer equipped with diode violet and blue lasers emitting at 405 nm and 488 nm, respectively. The side and forward scattering signals were used to get rid of cell debris and cell duplets (as shown in §10). Blue and green fluorescence coming from single cells were collected. The blue fluorescence intensity corresponded to the fluorescence of capsules loaded with DEX-blue associated with HeLa cells. Green fluorescence corresponded to the expressed GFP. Each measurement was repeated 3 times. From the blue fluorescence intensity distributions (analogues to the one shown in **Figure S23**) the mean blue fluorescence intensity per cell $\langle I_{blue} \rangle$ and the corresponding standard deviation were calculated and plotted versus the incubation conditions (**Figures S38 to S41**) for the different types of capsules.

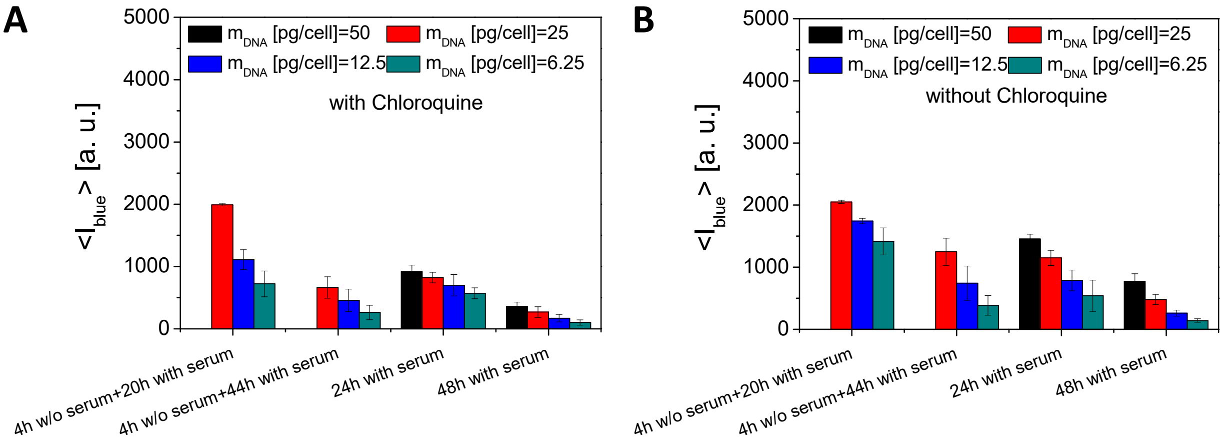

Figure S38. Mean blue fluorescence intensity $\langle I_{blue} \rangle$ of HeLa cells after incubation with DNA and DEX-blue co-loaded SiO_2 (low TEOS) capsules at different conditions of incubation. About 10,000 events were analyzed. The error bars correspond to the deviation from 3 independent experiments. $m_{DNA}/m_{DEX\text{-}blue} \approx 50/18 \approx 2.7$ in the capsules.

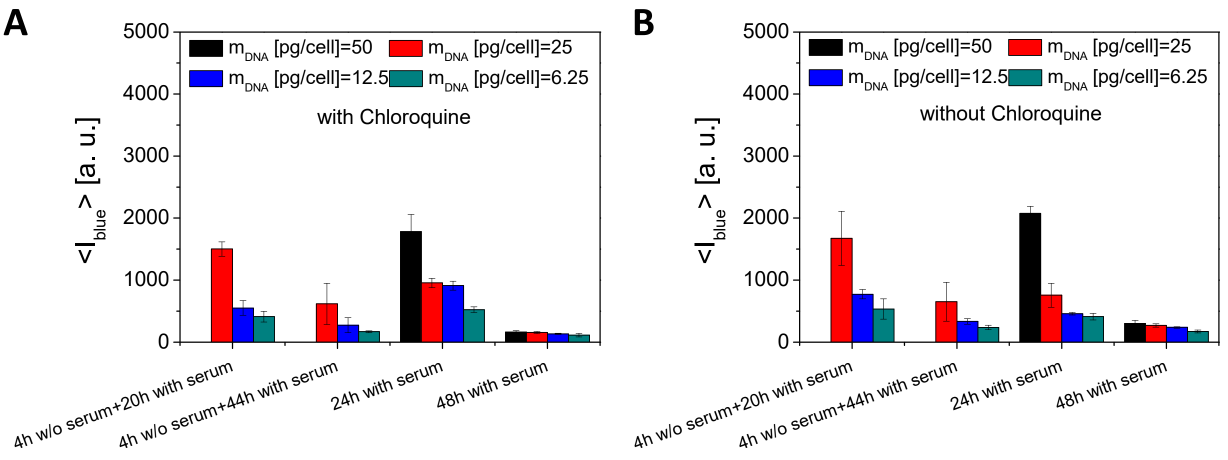

Figure S39. Mean blue fluorescence intensity <I_{blue}> of HeLa cells after incubation with DNA and DEX-blue co-loaded SiO_2/PARG (high TEOS) capsules at different conditions of incubation. About 10,000 events were analyzed. The error bars correspond to the deviation from 3 independent experiments. $m_{DNA}/m_{DEX\text{-}blue} \approx 50/15 \approx 3.4$ in the capsules.

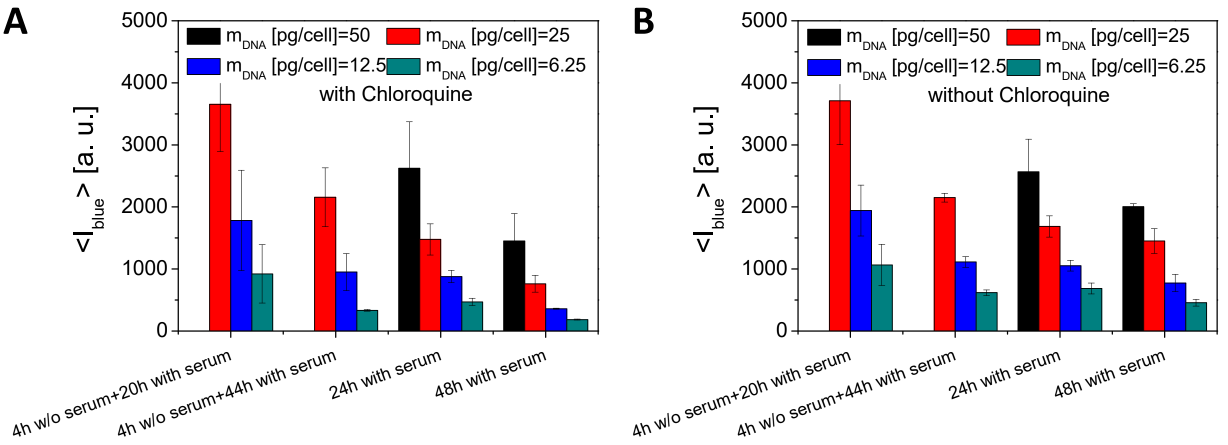

Figure S40. Mean blue fluorescence intensity <I_{blue}> of HeLa cells after incubation with DNA and DEX-blue co-loaded $(DEXS/PARG)_4$ capsules at different conditions of incubation. About 10,000 events were analyzed. The error bars correspond to the deviation from 3 independent experiments. $m_{DNA}/m_{DEX\text{-}blue} \approx 50/13 \approx 3.9$ in the capsules.

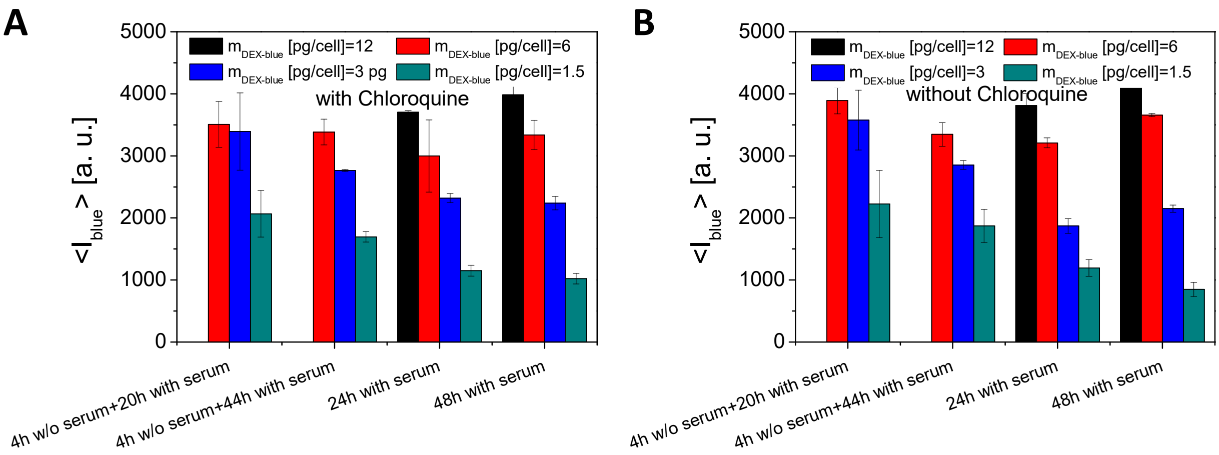

Figure S41. Mean blue fluorescence intensity <I_{blue}> of HeLa cells after incubation with DNA and DEX-blue co-loaded $(PSS/PAH)_4$ capsules at different conditions of incubation. About 10,000 events were analyzed. The error bars correspond to the deviation from 3 independent experiments. The $(PSS/PAH)_4$ capsules cannot be dissolved with pronase and, thus, the amount of encapsulated DNA could not be determined. The amount of added $(PSS/PAH)_4$ capsules refers to the amount of loaded DEX-blue.

At the same time, green fluorescence coming from cells, due to expression of GFP inside cells, was recorded with flow cytometry. Afterwards, the percentage T of transfected cells was determined. Each event in which the green fluorescence signal was above the green fluorescence signal of untreated cells was considered as transfected cell. The results are shown in **Figures S42 - S45** for the different types of capsules.

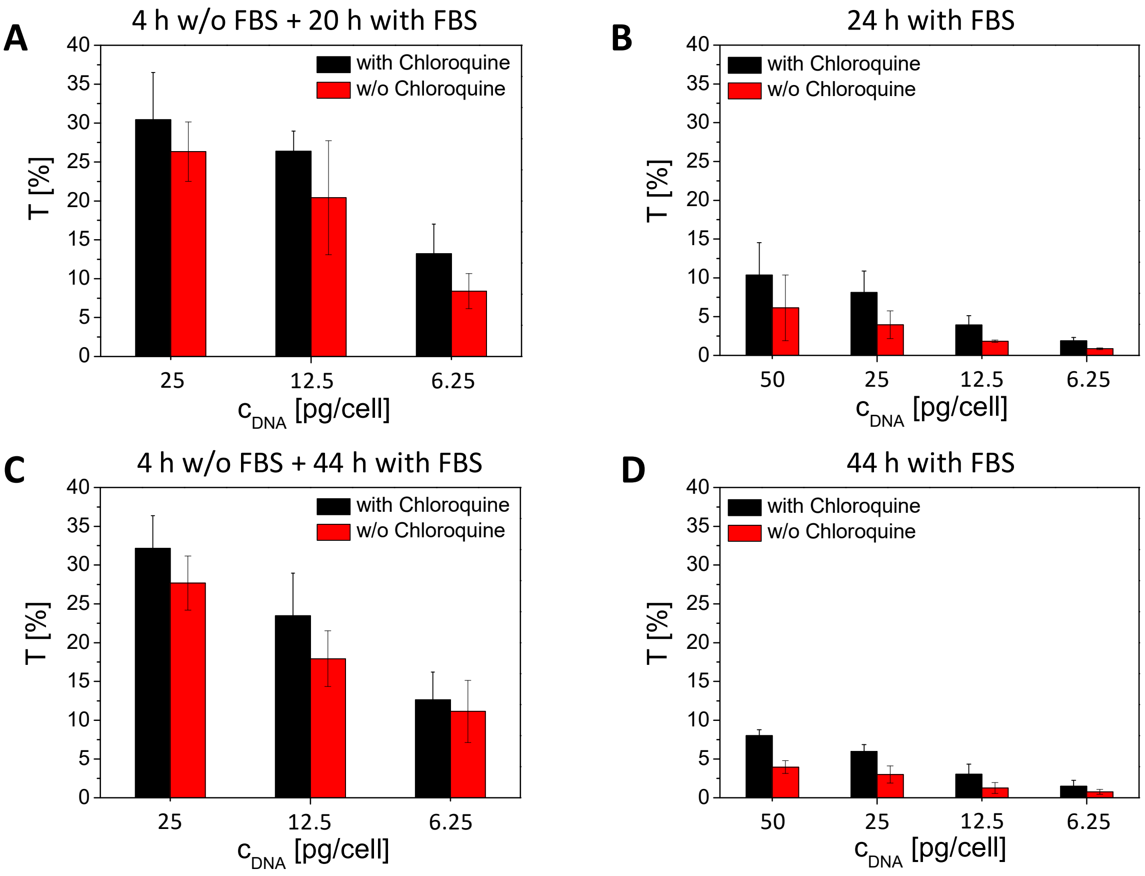

Figure S42. Transfection efficiency of HeLa cells by SiO_2 (low TEOS) capsules. Cells were incubated with capsules at different DNA concentrations in presence of chloroquine (black bars) and without chloroquine (red bars). Incubation times were A. 4 h in cell culture medium without FBS and then 20 h in cell culture medium with FBS; B. 24 h in cell culture medium with FBS; C. 4 h in cell culture medium without FBS and then 44 h in cell culture medium with FBS; D. 48 h in cell culture medium with FBS.

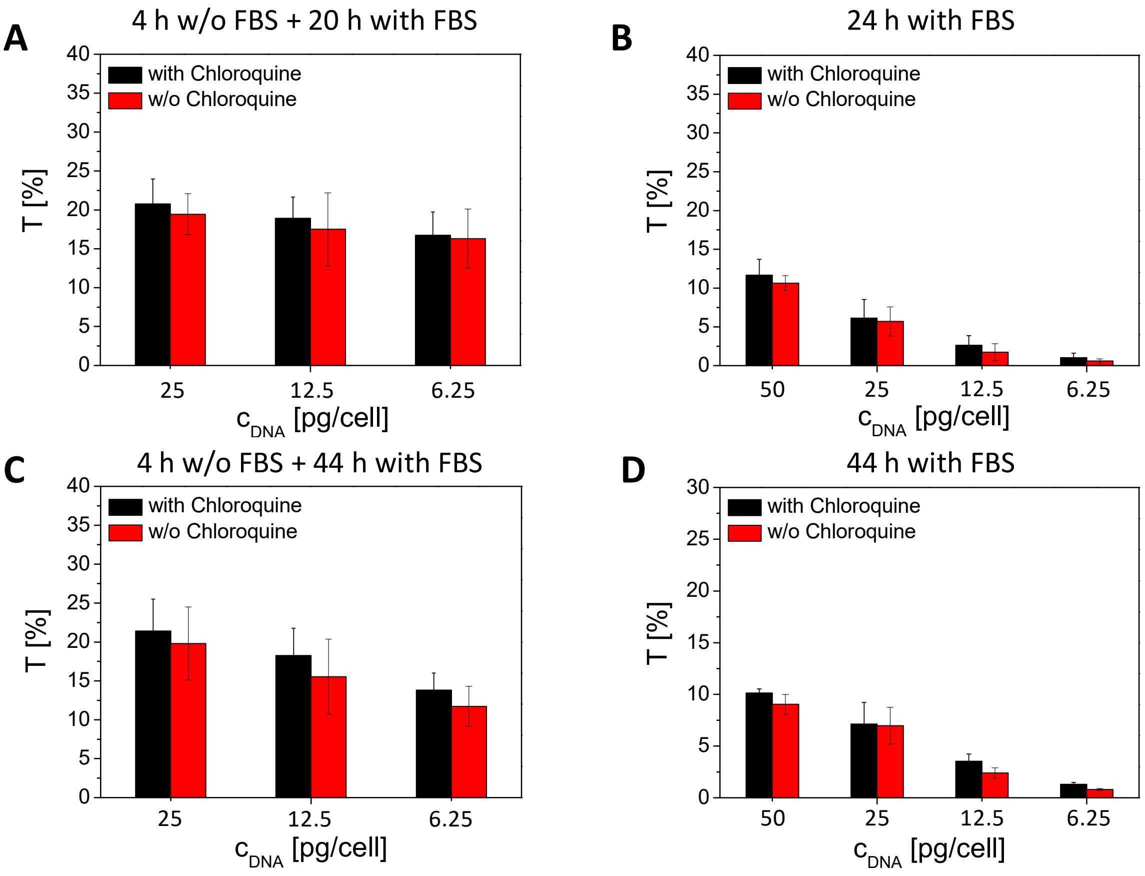

Figure S43. Transfection efficiency of HeLa cells by SiO_2 (high TEOS) capsules. Cells were incubated with capsules at different DNA concentrations in presence of Chloroquine (black bars) and without chloroquine (red bars). Incubation times were A. 4 h in cell culture medium without FBS and then 20 h in cell culture medium with FBS; B. 24 h in cell culture medium with FBS; C. 4 h in cell culture medium without FBS and then 44 h in cell culture medium with FBS; D. 48 h in cell culture medium with FBS.

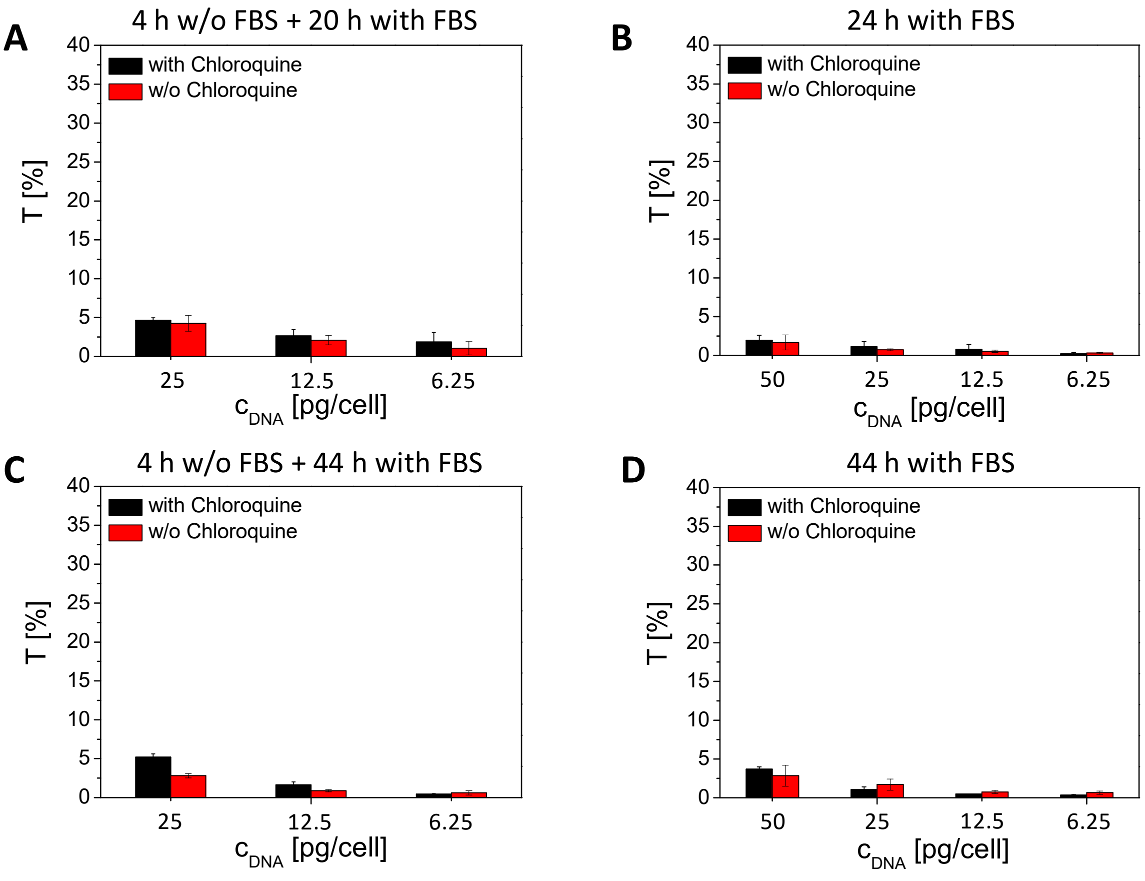

Figure S44. Transfection efficiency of HeLa cells by $(DEXS/PARG)_4$ capsules. Cells were incubated with capsules at different DNA concentrations in presence of chloroquine (black bars) and without chloroquine (red bars). Incubation times were A. 4 h in cell culture medium without FBS and then 20 h in cell culture medium with FBS; B. 24 h in cell culture medium with FBS; C. 4 h in cell culture medium without FBS and then 44 h in cell culture medium with FBS; D. 48 h in cell culture medium with FBS.

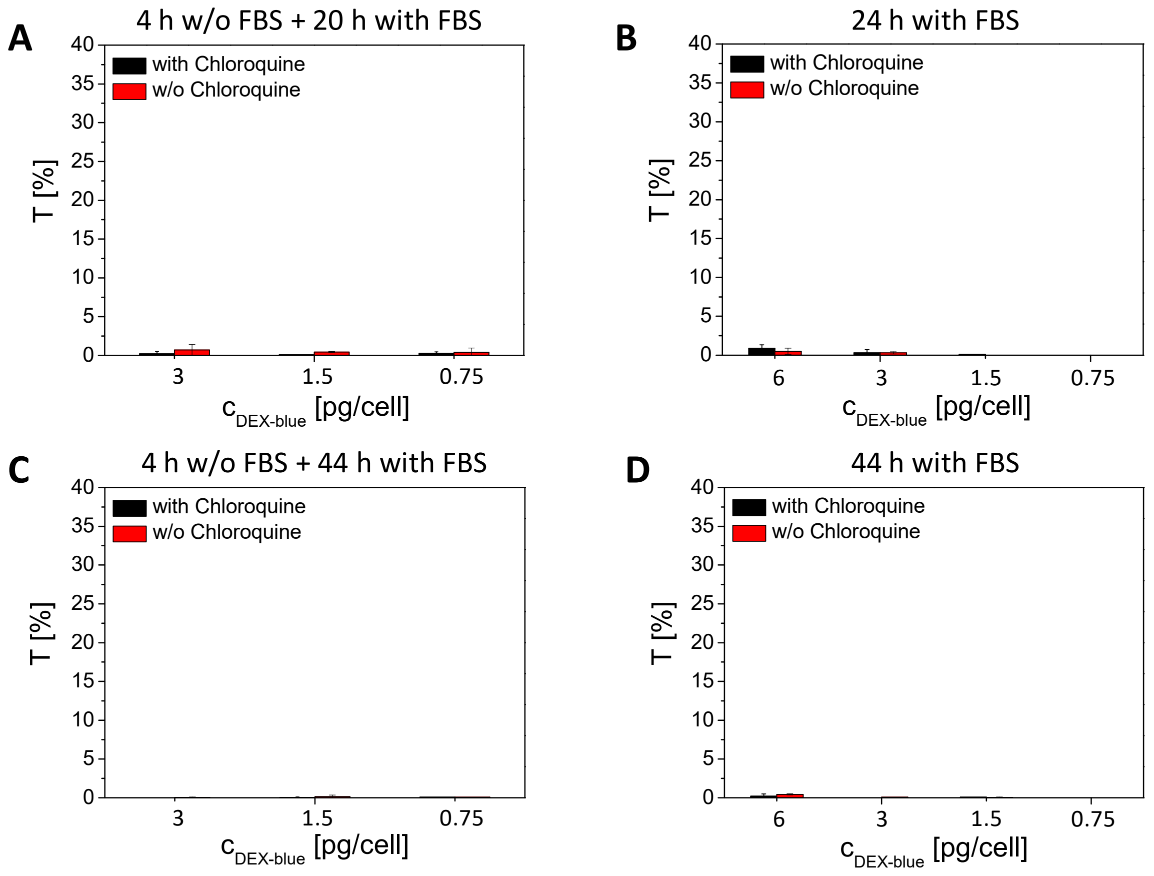

Figure S45. Transfection efficiency of HeLa cells by (PSS/PAH)$_4$ capsules. Cells were incubated with capsules at different DNA concentrations in presence of chloroquine (black bars) and without chloroquine (red bars). Incubation times were A. 4 h in cell culture medium without FBS and then 20 h in cell culture medium with FBS; B 24 h in cell culture medium with FBS; C. 4 h in cell culture medium without FBS and then 44 h in cell culture medium with FBS; D. 48 h in cell culture medium with FBS. As the DNA concentration in capsules could not be determined the capsule concentration is given as concentration of added DEX-blue.

15. References

1. Sukhorukov, G.B.; Donath, E.; Lichtenfeld, H.; Knippel, E.; Knippel, M.; Budde, A.; Mohwald, H. Layer-by-layer self assembly of polyelectrolytes on colloidal particles. *Colloids And Surfaces A-Physicochemical And Engineering Aspects* **1998**, *137*, 253-266.
2. Ott, A.; Yu, X.; Hartmann, R.; Rejman, J.; Schütz, A.; Ochs, M.; Parak, W.J.; Carregal Romero, S. Light-addressable and degradable silica capsules for delivery of molecular cargo to the cytosol of cells. *Chem. Mater.* **2015**, *27*, 1929–1942.
3. Trushina, D.B.; Bukreeva, T.V.; Antipina, M.N. Size-Controlled Synthesis of Vaterite Calcium Carbonate by the Mixing Method: Aiming for Nanosized Particles. *Crystal Growth & Design* **2016**, *16*, 1311-1319, doi:10.1021/acs.cgd.5b01422.

4. Bahrom, H.; Goncharenko, A.A.; Fatkhutdinova, L.I.; Peltek, O.O.; Muslimov, A.R.; Koval, O.Y.; Eliseev, I.E.; Manchev, A.; Gorin, D.; Shishkin, I.I., et al. Controllable Synthesis of Calcium Carbonate with Different Geometry: Comprehensive Analysis of Particle Formation, Cellular Uptake, and Biocompatibility. *ACS Sustainable Chemistry & Engineering* **2019**, *7*, 19142-19156, doi:10.1021/acssuschemeng.9b05128.
5. Zyuzin, M.V.; Díez, P.; Goldsmith, M.; Carregal-Romero, S.; Teodosio, C.; Rejman, J.; Feliu, N.; Escudero, A.; Almendral, M.J.s.; Linne, U., et al. Comprehensive and Systematic Analysis of the Immunocompatibility of Polyelectrolyte Capsules. *Bioconjugate Chem.* **2017**, *28*, 556–564.
6. Schneider, G.; Decher, G. Functional core/shell nanoparticles via layer-by-layer assembly. investigation of the experimental parameters for controlling particle aggregation and for enhancing dispersion stability. *Langmuir* **2008**, *24*, 1778-1789.
7. Boonacker, E.; Van Noorden, C.J.F. Enzyme Cytochemical Techniques for Metabolic Mapping in Living Cells, with Special Reference to Proteolysis. *J. Histochem. Cytochem.* **2001**, *49*, 1473-1486, doi:10.1177/002215540104901201.
8. Rivera_Gil, P.; Koker, S.D.; Geest, B.G.D.; Parak, W.J. Intracellular processing of proteins mediated by biodegradable polyelectrolyte capsules. *Nano Lett.* **2009**, *9*, 4398-4402.
9. Ma, X.; Hartmann, R.; Aberasturi, D.J.d.; Yang, F.; Soenen, S.J.H.; Manshian, B.B.; Franz, J.; Valdeperez, D.; Pelaz, B.; Feliu, N., et al. Colloidal Gold Nanoparticles Induce Changes in Cellular and Subcellular Morphology. *ACS Nano* **2017**, *11*, 7807–7820.
10. O'Brien, J.; Wilson, I.; Orton, T.; Pognan, F.o. Investigation of the Alamar Blue (resazurin) fluorescent dye for the assessment of mammalian cell cytotoxicity. *Eur. J. Biochem.* **2000**, *267*, 5421-5426.
11. Yang, F.; Riedel, R.; Pino, P.d.; Pelaz, B.; Said, A.H.; Soliman, M.; Pinnapireddy, S.R.; Feliu, N.; Parak, W.J.; Bakowsky, U., et al. Real-time, label-free monitoring of cell viability based on cell adhesion measurements with an atomic force microscope. *Journal of Bionanotechnology* **2017**, *15*, 23.
12. Semmling, M.; Kreft, O.; Muñoz Javier, A.; Sukhorukov, G.B.; Käs, J.; Parak, W.J. A Novel Flow-Cytometry-based Assay for Cellular Uptake Studies of Polyelectrolyte Microcapsules. *Small* **2008**, *4*, 1763-1768.
13. Hartmann, R.; Weidenbach, M.; Neubauer, M.; Fery, A.; Parak, W.J. Stiffness-Dependent In Vitro Uptake and Lysosomal Acidification of Colloidal Particles. *Angew. Chem.-Int. Edit.* **2015**, *54*, 1365-1368, doi:10.1002/anie.201409693.
14. Ashraf, S.; Said, A.H.; Hartmann, R.; Assmann, M.-A.; Feliu, N.; Lenz, P.; Parak, W.J. Quantitative particle uptake by cells as analyzed by different methods. *Angew. Chem.* **2020**, *59*, 5438-5453.
15. Yang, H.; Ganguly, A.; Cabral, F. Inhibition of cell migration and cell division correlates with distinct effects of microtubule inhibiting drugs. *The Journal of biological chemistry* **2010**, *285*, 32242-32250, doi:10.1074/jbc.M110.160820.
16. Kastl, L.; Sasse, D.; Wulf, V.; Hartmann, R.; Mircheski, J.; Ranke, C.; Carregal-Romero, S.; Martínez-López, J.A.; Fernández-Chacón, R.; Parak, W.J., et al. Multiple internalization pathways of polyelectrolyte multilayer capsules into mammalian cells. *ACS Nano* **2013**, *7*, 6605-6618, doi:10.1021/nn306032k.
17. Nazarenus, M.; Zhang, Q.; Soliman, M.G.; del Pino, P.; Pelaz, B.; Carregal_Romero, S.; Rejman, J.; Rothen-Ruthishauser, B.; Clift, M.J.D.; Zellner, R., et al. In vitro Interaction of Colloidal Nanoparticles with Mammalian Cells: What Have We Learned Thus Far? *Beilstein Journal of Nanotechnology* **2014**, *5*, 1477-1490.
18. Chanana, M.; Rivera Gil, P.; Correa-Duarte, M.A.; Parak, W.J.; Liz-Marzán, L.M. Physicochemical properties of protein-coated gold nanoparticles in biological fluids and cells before and after proteolytic digestion. *Angew. Chem. Int. Ed.* **2013**, *52*, 4179-4183, doi:10.1002/anie.201208019.
19. Tycko, B.; Maxfield, F.R. Rapid Acidification of Endocytic Vesicles Containing Alpha-2-Macroglobulin. *Cell* **1982**, *28*, 643-651.
20. Hu, Y.-B.; Dammer, E.B.; Ren, R.-J.; Wang, G. The endosomal-lysosomal system: from acidification and cargo sorting to neurodegeneration. *Translational Neurodegeneration* **2015**, *4*, 18, doi:10.1186/s40035-015-0041-1.

21. Rivera Gil, P.; Nazarenus, M.; Ashraf, S.; Parak, W.J. pH sensitive capsules as intracellular optical reporters for monitoring lysosomal pH changes upon stimulation. *Small* **2012**, *8*, 943-948.
